# Biogenesis of circular RNAs in vitro and in vivo from the *Drosophila Nk2.1*/*scarecrow* gene

**DOI:** 10.1093/g3journal/jkaf055

**Published:** 2025-03-12

**Authors:** Hyunjin Jeong, Suhyeon Son, Gyunghee Lee, Jae H Park, Siuk Yoo

**Affiliations:** Department of Life Sciences, Yeungnam University, Gyeongsan, Gyeongbuk 38541, Republic of Korea; Department of Life Sciences, Yeungnam University, Gyeongsan, Gyeongbuk 38541, Republic of Korea; Department of Biochemistry, Cellular and Molecular Biology, University of Tennessee, Knoxville, TN 37996, USA; Department of Biochemistry, Cellular and Molecular Biology, University of Tennessee, Knoxville, TN 37996, USA; Graduate Program of Genome Science & Technology, University of Tennessee, Knoxville, TN 37996, USA; Department of Life Sciences, Yeungnam University, Gyeongsan, Gyeongbuk 38541, Republic of Korea

**Keywords:** *Nk2.1/scarecrow*, circular RNA, intronic complementary sequence, back-splicing, age-dependent expression

## Abstract

The *scarecrow* (*scro*) gene encodes a fly homolog of mammalian *Nkx2.1*, which is vital for early fly development and for optic lobe development. Previously, *scro* was reported to produce a circular RNA in addition to traditional mRNAs. In this study, we report 12 different *scro* circular RNAs, which are either mono or multiexonic forms. The most abundant ones are circScro(2) carrying the second exon (E2) only and bi-exonic circScro(3,4) having both the third (E3) and fourth exon (E4). Levels of circScro(2) show an age-dependent increase in adult heads, supporting a general trend of high accumulation of circular RNAs in aged fly brains. In silico analysis of the introns flanking circular RNA exons predicts 2 pairs of intronic complementary sequences; 1 pair residing in introns 1 and 2 and the other in introns 2 and 4. The first pair was demonstrated to be essential for the circScro(2) production in cell-based assays; furthermore, deletion of the region including intronic complementary sequence components in the intron-2 reduces in vivo production of both circScro(2) and circScro(3,4) by 80%, indicating them to be essential for the biogenesis of the 2 circular RNAs. Besides the intronic complementary sequence, the intron regions immediately abutting exons seem to be responsible for a basal level of circular RNA formation. Moreover, ectopic intronic complementary sequence derived from the *laccase2* locus is comparably effective in circScro production, buttressing the importance of the hairpin loop structure formed by intronic complementary sequence for the biogenesis of circular RNA. Last, overexpressed *scro* alters outcomes of both linear and circular RNAs from the endogenous *scro* locus, suggesting that Scro plays a direct or indirect role in regulating the expression levels of either or both forms.

## Teaser

*Drosophila scarecrow (scro)* encoding a homeodomain is essential for nervous system development. This study reported that the circular form of RNAs is abundantly expressed from the endogenous *scro* locus. The formation of hairpin structures from intronic sequences is critical for the biogenesis of circular RNAs. Furthermore, the amount of *scro* circular RNA increases in an age-dependent manner, implying a role of *scro* in senescence. Although research on circular RNA has been actively performed recently, many aspects of its function are still uncovered. Therefore, the research of Jeong *et al*. will serve as a cornerstone for elucidating the biological function of circular RNA.

## Introduction

The circular RNAs (circRNAs) were first discovered in plant viroids and later in almost all organisms from archaea to humans, signifying them as common transcriptional products as linear RNA forms (linRNAs) (Sanger *et al*. 1976; Memczak *et al*. 2013; Ashwal-Fluss *et al*. 2014; Westholm *et al*. 2014). Interestingly, an abundance of circRNAs varies between tissues, and their expression patterns do not necessarily match with the mRNAs derived from the same locus, suggesting that the molecular mechanisms governing the biogenesis of circRNAs have divergently evolved from those of linRNAs (Salzman *et al*. 2013).

In eukaryotes, the circRNA is formed through a covalent bond between a 5′ splice donor and an upstream 3′ splice acceptor, an event known as “back-splicing” (Jeck *et al*. 2013; Jeck and Sharpless 2014; Wang *et al*. 2014; Li *et al*. 2018; Kristensen *et al*. 2019; Patop *et al*. 2019). Such a nonconventional splicing event is shown to be facilitated by a hairpin structure formed by base-pairing between so-called “intronic complementary sequences (ICSs)” found in the introns flanking the circRNA-coding exon(s) (Dubin *et al*. 1995; Ashwal-Fluss *et al*. 2014; Liang and Wilusz 2014; Zhang *et al*. 2014; Ivanov *et al*. 2015; Kramer *et al*. 2015; Starke *et al*. 2015; Aktas *et al*. 2017; Knupp *et al*. 2021). However, the lack of conserved sequence motifs in the ICSs implies the uniqueness of individual ICSs and likely an absence of universal *trans*-acting factor(s) binding to ICSs.

Functionally, circRNAs add another layer of complexity to gene regulation. One of the known roles of circRNAs is to regulate gene expression via binding to thus sequestering microRNAs, a function referred to as “miRNA sponge” (Hansen *et al*. 2013; Memczak *et al*. 2013; Zheng *et al*. 2016). Some of them are shown to act as a “protein sponge” by binding to RNA-binding proteins (RBPs), while others contain short open-reading frames producing truncated proteins (reviewed in Kristensen *et al*. 2019). However, the biological roles of the majority of circRNAs await further investigations.

Genome-wide deep RNA sequencing has identified circRNAs from numerous genes in various cell and tissue types in *Drosophila* (Westholm *et al*. 2014). Notably, many circRNAs are derived from the brain tissue, suggesting nervous tissue-specific mechanisms regulating circularization and their certain role(s) in this tissue type (Ashwal-Fluss *et al*. 2014; Westholm *et al*. 2014). Interestingly, the circRNAs in the adult brain accumulate with aging perhaps because of their enhanced stability (Jeck and Sharpless 2014; Westholm *et al*. 2014; Barrett and Salzman 2016). In line with this, a circRNA derived from a *sulfateless* gene (circSfl) is upregulated in *insulin* gene mutants, and overexpression of the circSfl extends the lifespan of fruit flies, suggesting a role of accumulated circSfl in longevity (Weigelt *et al*. 2020). Therefore, accumulated circRNAs in aged brains might be associated with the changes in neural structure and/or function during aging. These findings have shown that *Drosophila* is an excellent model system for understanding biogenesis and in vivo roles of circRNAs in a gene-specific manner.

The *scro* gene encodes a transcription factor belonging to the NK-2 homeobox family, most members of which are well known to act in regional or cell-type specification in *Drosophila*. *scro* expression is detected predominantly in the central nervous system (CNS) and pharynx (Zaffran *et al*. 2000; Yoo *et al*. 2020). We also have shown that *scro-*null mutations cause lethality between late embryonic and early larval stages, indicating that this gene plays a vital function (Yoo *et al*. 2020). Broad expression of *scro* in the central brain, ventral nerve cord (VNC), and optic lobe in larval and adult stages suggests various neuronal roles in both developing and developed CNS (Yoo *et al*. 2020). One such role is to specify neuroblast identity during the optic lobe development as one of the temporal transcription factors (Wang *et al*. 2011; Konstantinides *et al*. 2022). Another known function is to specify the intermediate neural progenitors in the lineage of the dorsal-medial type-II neural stem cells, which eventually give rise to the neurons in the adult central complex (Tang *et al*. 2022). *scro* expression is also detected in a subset of dopaminergic neurons and many other unidentified neuronal groups in the adult central brain and VNC, suggesting this gene's functioning in the differentiated neurons (Yoo *et al*. 2020). It is likely to act as a negative transcription regulator, as transgenic expression of *scro* in the *Drosophila* circadian pacemaker neurons downregulates the *Pigment-dispersing factor* (*Pdf*) gene expression, an important clock-downstream factor controlling the circadian locomotor activity rhythms (Renn *et al*. 1999; Park *et al*. 2000; Nair *et al*. 2020).

The *scro* gene produces 4 mRNA isoforms via alternative splicing (Yoo *et al*. 2020). In this study, we identified 12 different *scro* circRNAs; quantitative analyses of their levels show that the most abundantly expressed forms are circScro(2) and circScro(3,4). We found putative ICSs and demonstrated that they are essential for the biogenesis of *scro* circRNAs in vivo and in vitro. We further addressed key features of the ICS that determine the outcome of circRNAs.

## Materials and methods

### Fly strains

Oregon-R (OR) was used as a wild-type control. The following transgenic lines were used: knock-in *scro* lines, *scro*^*ΔE2−EGFP*^, *scro*^*ΔE2−Gal4*^, *scro*^*ΔE3−EGFP*^, and *scro*^*ΔE3−Gal4*^, each of which replaces exon-2 or exon-3 with either *EGFP* or *Gal4* coding sequences (Yoo *et al*. 2020); *UAS-scro*^*HA*^ (Nair *et al*. 2020); *hs-Cre* lines (BDSC# 1092 and 34516); and *nos-Cas9* line (KDRC# 233). Flies were raised at 25°C in food vials containing 0.85% agarose, 3.75% sucrose, 3% yeast, 8.4% corn meal, 2% tegosept (a.k.a. methylparaben), and 1% molasses.

### RNA isolation and cDNA synthesis

To analyze the expression of *scro* circRNAs during development, total RNA was purified from various stages of wild-type using the RNeasy system (Qiagen) according to the manufacturer's protocol with minor modifications. Briefly, the samples were dissolved in 350 µL of RLT lysis buffer with 7 µL of 2 M dithiothreitol. Following centrifugation, the supernatant was mixed with 70% ethanol and transferred to the spin column. After washing the column with RW1 buffer and RPE buffer, RNAs were eluted with 30 µL RNase-free water. The concentration and purity of the RNAs were measured using MaestroNano Spectrophotometer (MaestroGen, MN-913). One microgram of total RNA was reverse-transcribed by using the ImProm-II Reverse Transcriptase (Promega). Three types of primers were used for the reverse transcription reaction: oligo d(T) for linear RNA and either a gene-specific primer or random hexamer for both linear and circRNA. The reaction was performed at 25°C for 5 min, followed by at 42°C for 60 min, and then terminated at 70°C for 15 min.

### RNase R treatment and reverse transcription-PCR

Total RNAs were treated with exoribonuclease R (RNase R, Epicentre, RNR07250) to destroy linRNAs. One microgram of RNA was incubated for 30 min at 37°C with 3 units of RNase R or mock-treated with distilled water, and then cDNA was synthesized by using a random hexamer as described above. To identify various types of circRNAs, PCR was performed as follows: a 20-µL reaction contained 2 µL of cDNA, 5 µL of GoTaq G2 Green Master Mix (Promega), and 200 nM of primers. The thermal cycling conditions were as follows: 95°C for 3 min (initial denaturation), followed by 40 cycles of 95°C for 1 min (denaturation), 60°C for 1 min (annealing), and 72°C for 1 min (extension) and then by 1 cycle of 72°C for 5 min (final extension).

### DNA constructs for testing ICS

For the transfection assay, a 905-bp fragment containing the second exon (E2) and flanking intronic sequences was amplified by PCR using the wild-type genomic DNA as a template and scro-I1-NoICS-F and scro-I2-NoICS-R primer set. The PCR product was cloned into *Bam*HI and *Kpn*I sites in the pPacPL vector (*Drosophila* Genomics Resource Center), generating the pNoICS backbone (Fig. 4Aa). To investigate the role of putative ICSs in circRNA production, a 179-bp within intron-1 and a 565-bp fragment within intron-2 were amplified by PCR using primer sets of scro-I1-BF/scro-I1-BR and scro-I2-KF/scro-I2-KR, respectively. These fragments were cloned into *Bam*HI and *Kpn*I sites in pNoICS. According to the orientations of these ICS fragments, we obtained 4 constructs, pS-ICS-FF, pS-ICS-FR, pS-ICS-RF, and pS-ICS-RR, as illustrated in Fig. 4Ab.

To test the effect of ICS originating from a different gene on circScro(2) expression, the *scro* ICSs were replaced by those from *laccase2* (Kramer *et al*. 2015). To do this, a 242-bp fragment within intron-1 and a 392-bp one within intron-2 were amplified by PCR using laccase2-I1-BF/laccase2-I1-BR and laccase2-I2-KF/laccase2-I2-KR primer sets, respectively, and then cloned into *Bam*HI and *Kpn*I site in the pNoICS. Four DNA constructs, pL-ICS-FF, pL-ICS-FR, pL-ICS-RF, and pL-ICS-RR, were shown in Fig. 4Ac.

To assess the effect of ICS lengths on the efficiency of *scro* circRNA formation, serial deletions of 21 bp from either 5′ or 3′ end of the 105-bp of ICS in intron-2 were made by employing a fusion-PCR strategy (Cha-Aim *et al*. 2012). For instance, to delete a 21-bp from the 5′ end, the first 2 PCRs were performed using pS-ICS-FF as a template, and 2 primer sets (scro-I2-KF/scro-I2-5′-Δa-R and scro-I2-5′-Δa-F/scro-I2-KR). Equimolar amounts of the 2 overlapping PCR products were mixed and used as a PCR template along with scro-I2-KF and scro-I2-KR primers. The resulting product was cloned into the *Kpn*I site of the pS-ICS-F plasmid carrying only *scro* intron-1 ICS (S-ICS1) to generate p5′-Δa construct (Fig. 5A). Similar approaches were used for other deletion constructs. All primers are listed in Supplementary Table 1.

### Quantitative real-time PCR

To quantify the expression levels of linRNAs and circRNAs of *scro*, cDNA was synthesized with random hexamer as described above. Each PCR contained 1 µL of cDNA, 200 nM of each forward and reverse primer, and 10 µL of power SYBR green PCR master Mix (Promega). The real-time quantitative (RT-qPCR) was performed using a fluorescent quantitative detection system (FQD-96A, Bioer). The cycling conditions were 95°C for 2 min, followed by 40 cycles of 95°C for 15 s and 60°C for 1 min. Either *rp49* (*ribosomal protein 49*) or *act5C* (*actin 5C*) was used as a control for normalization. The relative expression levels of RNAs were evaluated by the ΔΔCt-based method using LineGene 9600 Plus software (Bioer) and showed the log10-fold difference in Figs. 3B and 4B. The experiments were repeated at least 3 times. PCR primers are shown in Supplementary Tables 2 and 3.

### Cell culture and transfection assay

Schneider's 2 (S2) cells were cultured in Schneider's Insect Medium (WELGENE, South Korea) supplemented with 10% fetal bovine serum (Cytiva, Pasching, Austria) and 1% penicillin/streptomycin (Cytiva). The cells were maintained in an incubator at 23°C and subcultured at 80–90% confluency (about once every week). To assess the effect of ICS on the formation of circRNAs, S2 cells were cultured in a 6-well plate to 60% confluence, transfected with 4 µg of plasmid DNA using the FuGENE HD Transfection (Promega) for 24 h, and then total RNA was isolated from the transfected cells for RT-qPCR.

### Generation of a *scro* mutation lacking ICS

To identify candidate ICSs within the *scro* introns, all intron sequences annotated in the Flybase (https://flybase.org/) were aligned using EMBOSS (https://emboss.sourceforge.net/apps/cvs/emboss/apps/einverted.html) with the following parameters: ICS for circScro(2) with the Gap penalty = 40, the min score threshold = 150, the match score = 300, mismatch score = 30, and the max extent of repeats = 250; ICS for circScro(3,4) with the Gap penalty = 5, the min score threshold = 0, the match score = 150, mismatch score = −3, and the max extent of repeats = 150. For the analysis of ICS in *laccase2*, we used the parameters with the Gap penalty = 5, the min score threshold = 250, the match score = 169, mismatch score = 20, and the max extent of repeats = 475 options (Supplementary Fig. 2).

To delete the 1,180-bp region containing ICS from the intron-2, we employed a CRISPR/Cas9-mediated genome editing system. The target cleavage sites were selected using the flyCRISPR Optimal Target Finder tool (Gratz *et al*. 2014; https://flycrispr.org/). Two complementary sets of gRNA oligonucleotides (Supplementary Table 1), ΔI2-ICS-gRNA1-S/ΔI2-ICS-gRNA1-AS and ΔI2-ICS-gRNA2-S/ΔI2-ICS-gRNA2-AS, were annealed, cloned into the pU6-Bbs-chiRNA at *Bbs*I site, and then injected into *nos-Cas9* embryos. Approximately 50 G0 flies were individually crossed with *y w*, and 10 G1 flies from each G0 line were singly mated with *y w*; *Sb*/*TM6B* balancer stock. After 4 days, G1 flies were screened by single-fly PCR using a primer set I2F/I2R (Supplementary Table 2); the deletion line (ΔI2-ICS) is expected to produce a 351-bp PCR product, instead of the 1,531-bp fragment from the wild type. Since the positive G1 flies are heterozygous for the ΔI2-ICS allele, positive G2/TM6B flies were crossed to the balancer to establish the stocks (Supplementary Fig. 3). As a result, we established 2 lines, namely *ΔI2-ICS-44* and *ΔI2-ICS-45*, which carry the genomic lesion confirmed by sequencing (Fig. 6A).

### Statistical analysis

The qPCR experiments in this study were independently repeated for at least 3 times, and the results (bar graphs) were presented as the mean ± standard deviation (SD). Unpaired 2-tailed Student's *t*-tests were performed to detect any significant difference between the 2 groups using Microsoft Excel software (version 2203). *P*-values <0.05 were considered significant.

## Results

### Verification of circRNAs from *scro* transcripts

**Fig. 1.**
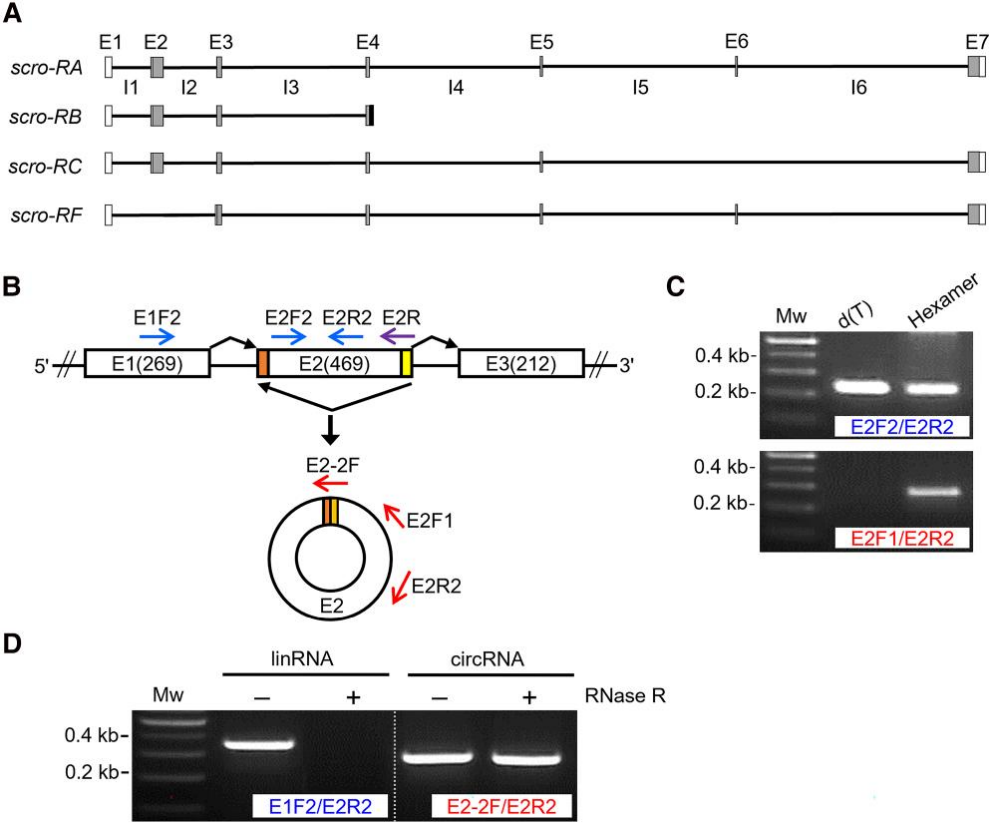
Verification of a circRNA derived from the *scro* locus. A) Exon (E)–intron (I) organization of the *scro* locus containing 8 exons (E1–E7 and *RB*-specific exon). According to FlyBase, the 4 mRNA isoforms are generated by alternative splicing. White boxes, UTRs; gray boxes, coding regions; black box, *RB*-specific exonic sequence; otherwise, it is an intronic region for the others. B) Schematic diagram of RNA splicing leading to the generation of circScro(2). The noncanonical exon junction point is formed when the 5′ splice site of E2 (yellow) is joined to the upstream 3′ splice site (orange) via back-splicing. E2R primer (violet arrow) was used for cDNA synthesis, and the convergent primers (blue arrows) and divergent primers (red arrows) were used for PCR. C) RT-PCR results using E2F2/E2R2 primer set (upper panel) and E2F1/E2R2 primer set (lower panel). The primers used for cDNA synthesis are shown above for each lane. PCR fragments were amplified using divergent primers in cDNA samples synthesized from random hexamer, but not in cDNAs from oligo d(T) primer (lower panel), indicating the presence of circScro(2). D) Effect of RNase R treatment on the detection of circRNA. Total RNA samples were either mock-treated (−) or RNase R-treated (+) before the cDNA synthesis using hexamer. In RNase R-treated RNA samples, only cDNAs using exon junction primer (E2-2F) were amplified, verifying the existence of *scro* circRNAs. Mw represents a 100-bp DNA ladder.

The *scro* is annotated to produce 4 linear transcripts (*RA*, *RB*, *RC*, and *RF*) via alternative splicing (Fig. 1A). It was also reported that a circRNA carrying a single exon originated from this locus (Westholm *et al*. 2014). Since the *scro* locus contains several large introns, it possibly produces more than one type of circRNA. To obtain a more comprehensive feature of the *scro* circRNA isoforms, total RNAs were extracted from wild-type adult heads where *scro's* linear transcripts are most abundant (Yoo *et al*. 2020). We designed a set of convergent PCR primers (E2F2 and E2R2) that are intended to detect both linear and circular forms and a set of divergent primers (E2F1 and E2R2) that should detect only circular forms (Fig. 1B). When we tested oligo d(T)-primed cDNA samples that represent linRNAs, convergent primers generated PCR product [Fig. 1C lane d(T) in upper panel] but divergent primers did not [Fig. 1C, lane d(T) in lower panel]. However, with hexamer-primed cDNA samples, PCR products were generated by both convergent and divergent primers (Fig. 1C), indicating the presence of a circRNA from E2; this was confirmed by sequencing and referred to as circScro(2) according to the recommended nomenclature of the circRNAs (Chen *et al*. 2023).

To ascertain the foregoing result, total RNAs were treated with RNase R to digest linear RNAs before the cDNA synthesis, and then PCR was carried out using either convergent primers (E1F2 and E2R2) for linRNAs or junction and divergent primers (E2-2F and E2R2) for circRNAs (Fig. 1B). As expected, linRNA-targeting primers produced PCR product from mock-treated RNA, but none from the RNase R-treated one (Fig. 1D, left panel). In contrast, circRNA-targeting primers produced PCR products from both samples (Fig. 1D, right panel). The results validate our approach being effective in finding exonic *scro* circRNAs.

### Identification of 12 different circRNA forms from *scro*

**Fig. 2.**
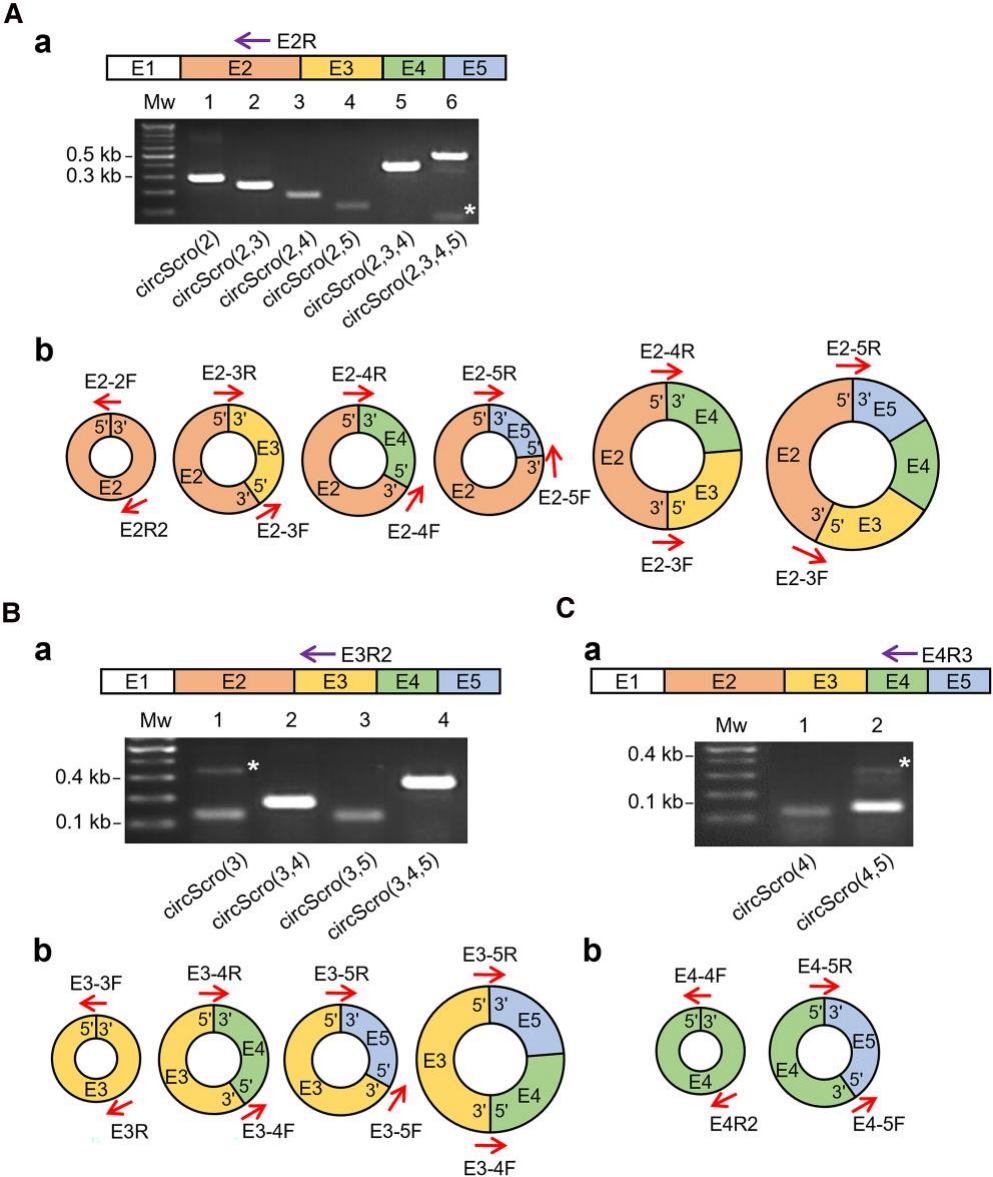
Identification of different types of *scro* circRNAs. To detect various types of exonic circRNAs, cDNAs were synthesized from RNase R-treated RNAs using gene-specific primers (violet arrows above agarose gels), and RT-PCR was performed using the junction primers spanning the exon–exon joining regions (red arrows in diagrams). In gel images, nonspecific bands are indicated by asterisks, and Mw represents a 100-bp DNA ladder. Schematic diagrams represent *scro* circRNAs carrying 1 to 4 exons. Each exon is differently color coded. A) Six types of circRNAs detected from E2R-primed cDNA samples are shown in agarose gel Aa) and diagrams Ab). The circRNA types corresponding to each lane are indicated at the bottom of the agarose gel. The sizes of PCR fragments are as follows: lane 1, 297 bp; lane 2, 242 bp; lane 3, 182 bp; lane 4, 126 bp; lane 5, 394 bp; and lane 6, 490 bp. B) Four types of circRNAs detected from E3R2-primed cDNA samples are shown in agarose gel Ba) and diagrams Bb). Lane 1, 146 bp; lane 2, 178 bp; lane 3, 122 bp; and lane 4, 274 bp. C) Two types of circRNAs detected from E4R3-primed cDNA samples are shown in agarose gel Ca) and diagrams Cb). Lane 1, 122 bp and lane 2, 123 bp.

Since circRNAs are often multiexonic, we wondered if there are additional E2-containing circRNAs. To test this, we designed 6 different sets of primers, as illustrated, each of which was intended to detect a specific combination of E2-containing multiexonic circRNAs (Fig. 2Ab). As a result, we identified 6 distinct PCR products using cDNAs generated from RNase R-treated RNA. In addition to circScro(2), sequencing of these products revealed 5 multiexonic circRNAs, which are referred to as circScro(2,3), circScro(2,4), circScro(2,5), circScro(2,3,4), and circScro(2,3,4,5), respectively (Fig. 2Aa; Supplementary Fig. 1A). These products lacked intronic region, verifying characteristic back-splicing events for all identified circRNAs. By extending this strategy to other exons, we identified 4 E3-containing circRNAs, circScro(3), circScro(3,4), circScro(3,5), and circScro(3,4,5) (Fig. 2Ba and b; Supplementary Fig. 1B), and 2 including the E4, circScro(4) and circScro(4,5) (Fig. 2Ca and b; Supplementary Fig. 1C). Despite our painstaking efforts, we were unable to find circRNAs including E1, E6, and E7. This is consistent with previous findings showing that the first or last exon is rarely made into circRNAs, presumably because they are flanked by only one intron (Salzman *et al*. 2012; Lasda and Parker 2014; Westholm *et al*. 2014; Gruner *et al*. 2016). However, it was surprising not to see circRNAs carrying E6, although this exon is flanked by the 2 largest introns. The circRNAs can be sorted into 3 types based on their intronic and/or exonic compositions: exonic circRNAs, intronic circRNAs, and exon-intron circRNAs (Lasda and Parker 2014; Shen *et al*. 2015). All 12 identified *scro* circRNAs are either single or multiexonic, and the production of the latter suggests that the interexonic introns are precisely excised out.

### Differential expression of *scro* circRNA isoforms

**Fig. 3.**
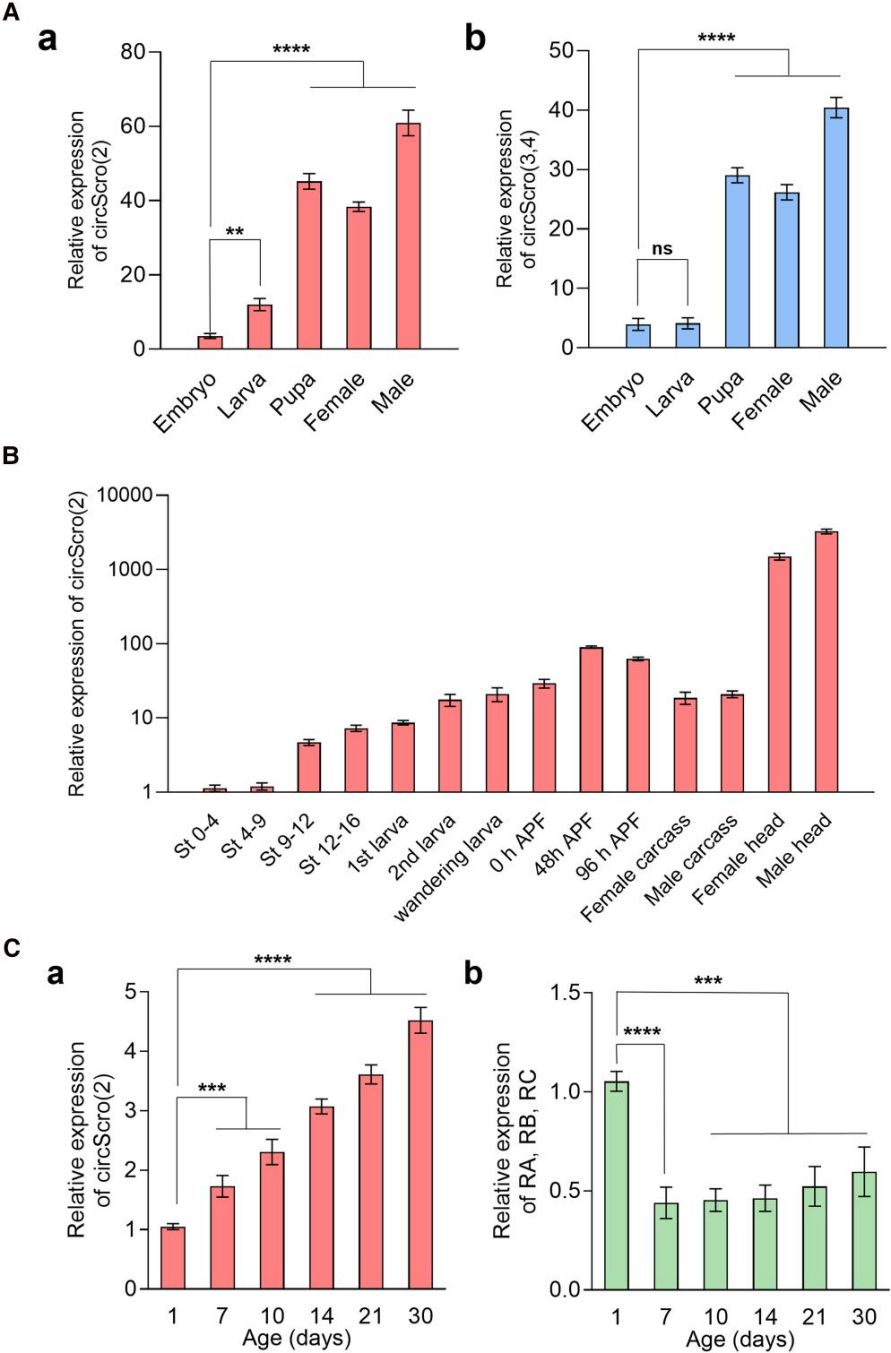
Expression levels of circRNAs during development and aging by RT-PCR. A) Expression levels of the 2 predominant circRNAs [circScro(2) and circScro(3,4)] at major developmental stages. The levels of other types of circRNAs were too low to measure by RT-PCR. For cDNA synthesis, the random hexamer was used. For PCR, E2-2F/E2R2 primer set was used for circScro(2) Aa) and E3-4F/E3-4R set for circScro(3,4) Ab). Each bar represents mean ± SD (*n* = 3). ns, not significant. **P < 0.05, ****P < 0.0001 compared with circScro(2) levels of embryo. B) Developmental expression profile of circScro(2). The random hexamer was used for cDNA synthesis, and E2-2F/E2R2 primers were used to detect circScro(2). Adult carcasses and heads were prepared from 5-day-old adults. The expression level from the circScro(2) at stage 0–4 embryos was used as a reference to calculate the relative levels of circRNAs at other stages. Each bar represents mean ± SD (*n* = 3). C) Aging-dependent expression levels of circScro(2) and linRNAs (*RA*, *RB*, and *RC*). The circRNA expression levels increase in an age-dependent manner Ca), while linRNA levels detected by E1F2/E2R2 primers decrease roughly by 50% within 7 days and onward Cb). Each bar represents mean ± SD (n = 3). ***P < 0.001, ****P < 0.0001 compared with 1-day-old adults.

Expression levels of each circRNA were assessed from different developmental stages by RT-qPCR. Expressions of circScro(2) and circScro(3,4) types were readily detectable from embryonic to adult stages (Fig. 3A). In general, both circRNAs increased during development, and the highest levels were found in adult males. Other circRNAs were weakly detected in all stages examined, indicating that circScro(2) and circScro(3,4) are the 2 most prevalent forms expressed throughout development and in the adult stage.

Next, we compared the levels of circRNA in more finely staged animals during development. We focused on the circScro(2) as it is the major form. Levels of circScro(2) were elevated up to the mid-pupal stage [48 h after puparium formation (APF)] and then decreased slightly at the late-pupal stage (Fig. 3B). As expected from the CNS being the main *scro* expression site (Yoo *et al*. 2020), the highest expression levels of circScro(2) were found in the adult heads (Fig. 3B).

Of interest, most of the *Drosophila* circRNAs detected in the brain were shown to accumulate with age (Westholm *et al*. 2014; Weigelt *et al*. 2020). This prompted us to examine whether circScro(2) expression also follows this trend. Indeed, the levels of circScro(2) increased nearly 4.5-fold over 30 days of aging, whereas combined linRNA levels of RA, RB, and RC amplified using E1F2 and E2R2 primers reduced by half within 7 days of aging and then remained flat afterward (Fig. 3C).

### Intronic sequences required for the biogenesis of *scro* circRNAs

ICSs located in introns flanking a circRNA exon or the first and last exons of multiexonic ones are known to play a critical role in the production of circRNAs, as they facilitate the formation of the stem-and-loop structure and then back-splicing of the paired introns to release the circRNAs (Ivanov *et al*. 2015; Wang *et al*. 2019). Potential ICSs were proposed in many *Drosophila* genes producing circRNAs (Ashwal-Fluss *et al*. 2014; Kramer *et al*. 2015; Weigelt *et al*. 2020), but their actual involvement in the circularization was validated only in a few genes such as *muscleblind* (*mbl*) and *laccase2* (Ashwal-Fluss *et al*. 2014; Kramer *et al*. 2015).

To find putative ICSs involved in the formation of circScro(2) and circScro(3,4), intronic sequences flanking exons of these circRNAs were examined for their complementarity, as described in the *Materials and Methods* section. As a result, we found 2 putative ICSs consisting of at least 80–105 nucleotides; one in intron-1 and intron-2 and the other in intron-2 and intron-4, which are possibly involved in the production of circScro(2) and circScro(3,4), respectively (Supplementary Fig. 2A and B).

**Fig. 4.**
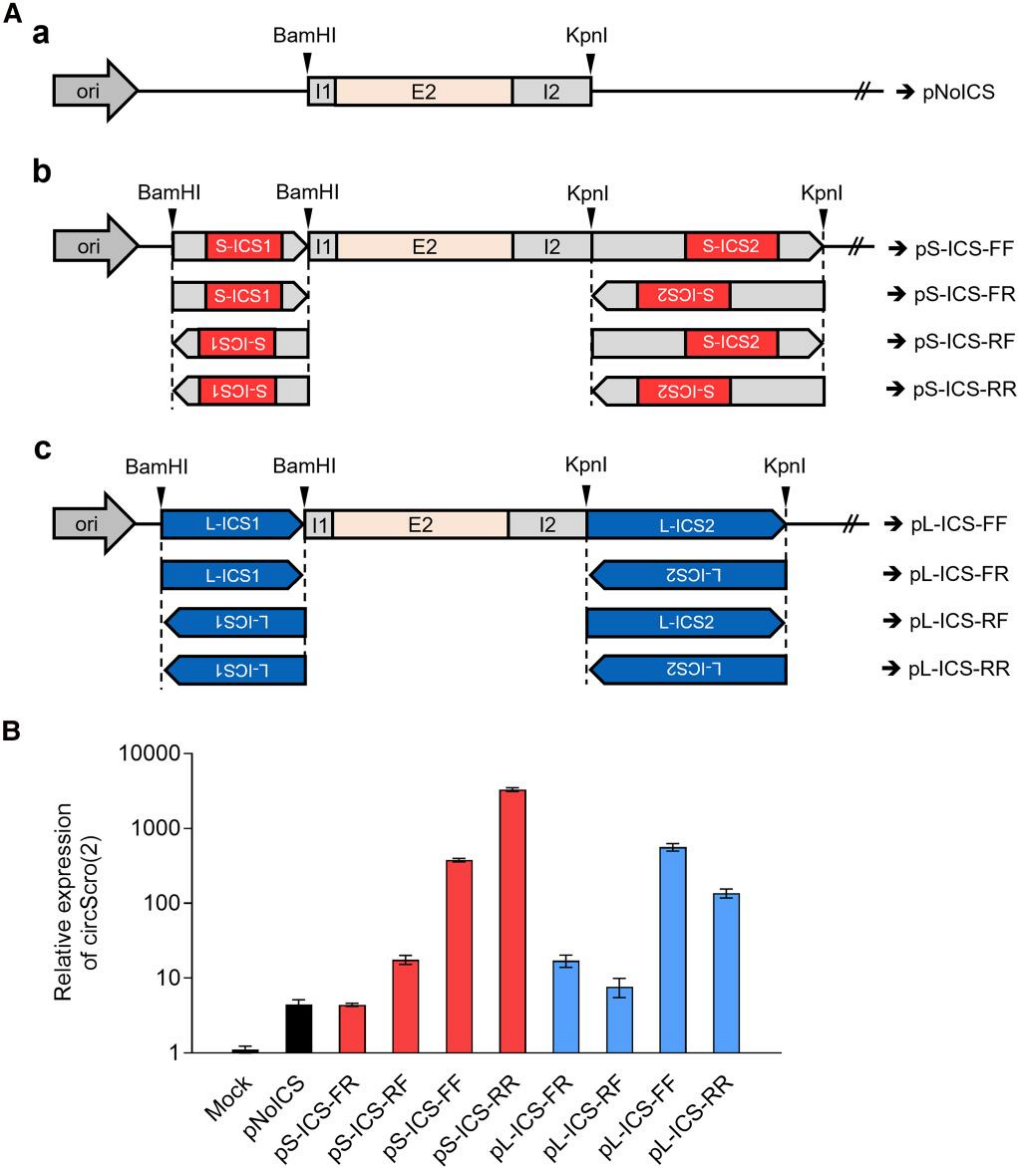
Effects of ICS orientation and origin on circScro(2) expression in vitro. A) Schematic diagrams of DNA constructs for transfection assay. Aa) pNoICS was generated by cloning of 905-bp PCR fragment carrying *scro* E2 and the flanking intronic sequences into the pPacPL vector. Ab) The 179-bp containing S-ICS1 and 565-bp containing S-ICS2 fragments were cloned into pNoICS at *Bam*HI and *Kpn*I sites, respectively, generating 4 constructs according to their orientations. The first letter following ICS designates the orientation of S-ICS1, and the second letter designates the orientation of S-ICS2. “F” stands for the same direction as genomic DNA, and “R” for reverse orientation. Ac) The 242-bp containing L-ICS1 and 392-bp containing L-ICS2 fragments were cloned into pNoICS at *Bam*HI and *Kpn*I sites, respectively, generating 4 constructs according to their orientations. B) RT-PCR results showing circScro(2) levels in transfected S2 cells. The expression levels of circScro(2) (red bars) in both pS-ICS-FF and pS-ICS-RR are much higher compared with those in pS-ICS-FR and pS-ICS-RF, strongly implying the importance of hairpin structure for the biogenesis of circRNA. Similar results were obtained when *scro* ICS was replaced with *laccase2* ICS (blue bars), indicating that the origin of ICS is not a crucial factor for circRNA formation. The qPCR was carried out using the E2-2F/E2R2 primer set, and each expression level was compared with the mock control. Each bar represents mean ± SD (*n* = 3).

We tested the first candidate ICS in cultured cells to see if they are indeed required for the biogenesis of the circScro(2). We first made a plasmid containing E2 and flanking intronic regions but excluding the putative ICS (pNoICS; Fig. 4Aa). Cells transfected with the pNoICS produced low levels of circScro(2), but significantly higher when compared with those with the blank plasmid (mock), indicating that the immediately flanking intronic regions are necessary for circularizing E2 albeit at low frequency (Fig. 4B). We consider this basal level expression. Next, extended intron-1 and intron-2 fragments including the 105-bp ICSs were tested in their natural orientations [pS-ICS-FF, shortly FF; “S” represents *scro* and “forward (F)” indicates the same orientation of ICS as in the *scro* genomic sequence]. Remarkably, the FF construct substantially increased circScro(2) levels, indicating that the ICS-mediated hairpin structure promotes circScro(2) (Fig. 4B). Since the intronic regions immediately flanking E2 are necessary for the basal level of circScro(2) production, both ICS and flanking regions are needed to give rise to full production of circScro(2).

To further confirm if the base-pairing of the ICSs is essential for the circularization, we tested reversely oriented ICS (RR) (Fig. 4Ab). Since both FF and RR can similarly make base-pairing in theory, we anticipated that the RR construct would facilitate circScro(2) production as comparably as FF one does. Intriguingly, the RR produced the circScro(2) at even higher levels than the FF did (Fig. 4B). We reasoned that closer proximity of *scro* intron-2 ICS (S-ICS2) to the E2 in the RR context might somehow promote the hairpin formation further. If this is the case, the distance between ICS and circularizing exon could be another factor that determines the efficacy of back-splicing. In contrast to FF and RR, circScro(2) expression from FR was at the basal level, while that from RF was slightly higher than FR (Fig. 4B). In summary, both base-pairing of ICS and their proximity to the exon determine the output levels of circRNAs.

Our next question is whether the formation of hairpin structure mediated by ICS, regardless of its origin, is sufficient for the biogenesis of *scro* circRNA. To test this, we employed the ICS found for a *laccase2* circRNA formation (Kramer *et al*. 2015; Supplementary Fig. 2C). As did for the S-ICS case, the *laccase2* ICS (L-ICS1 and L-ICS2) were inserted into pNoICS to generate pL-ICS-FF, -FR, -RF, and -RR (Fig. 4Ac). Both FF and RR produced significantly higher levels of circScro(2) than FR or RF did (Fig. 4B). The *laccase2* FF outcome was comparable twitho that of *scro* FF, while *laccase2* RR was less effective than *scro* RR. Taking these results together strongly supports that ICS-mediated hairpin formation is the key event for the production of circScro(2).

### Finding a core region of ICS essential for the productivity of circScro(2)

**Fig. 5.**
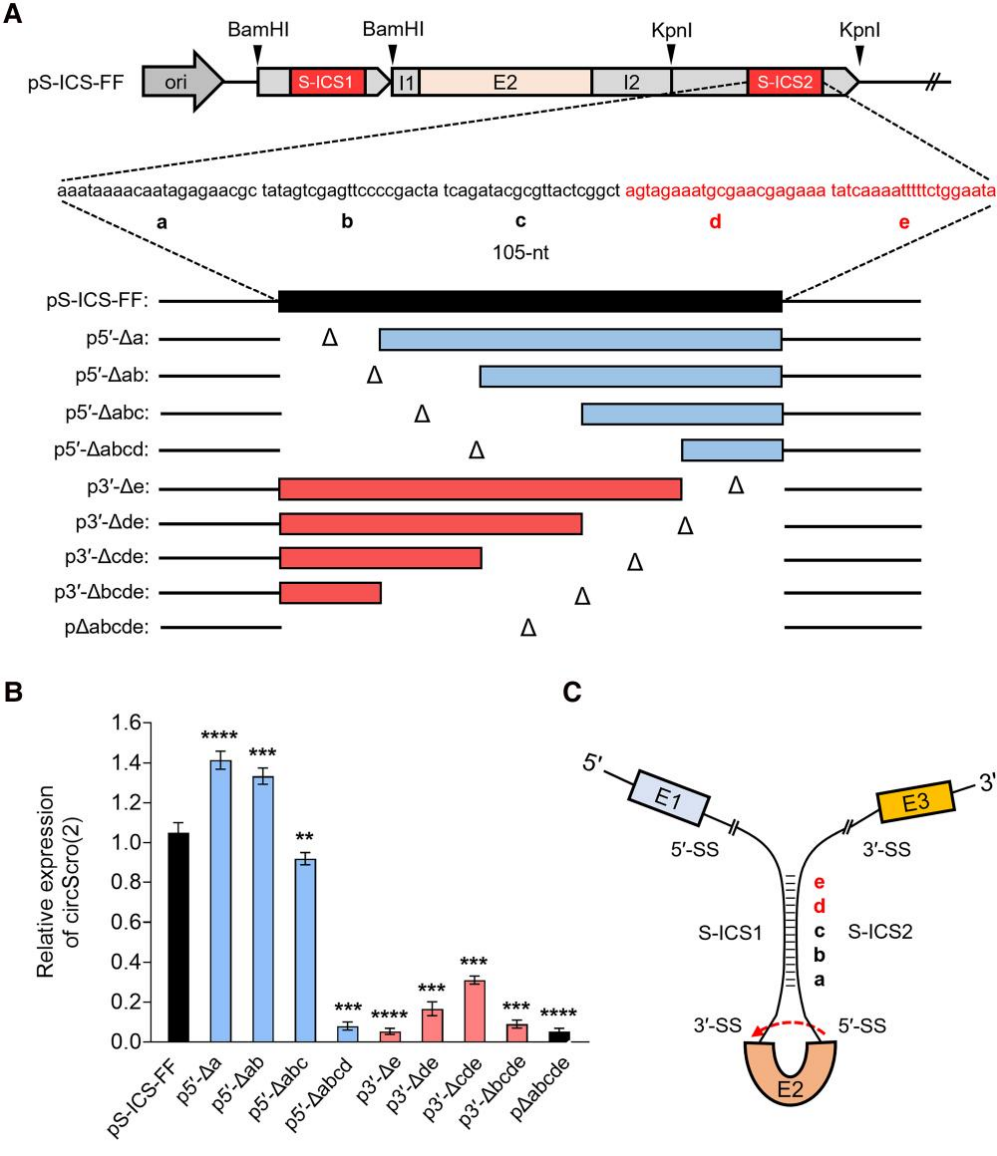
Effect of ICS length on circScro(2) expression. A) Schematic diagrams of the deletion constructs. The 105-nt of S-ICS2 was serially truncated by 21-nt as represented by “a” through “e”. B RT-PCR results of analyzing the expression levels of circScro(2) after transfection of each DNA construct into the S2 cell line. Primers used for cDNA synthesis and PCR were hexamer and E2-2F/E2R2, respectively. Comparable circScro(2) levels between p5′-Δabc and pS-ICS-FF (positive control) suggest that at least a 40-bp length of ICS is required for circScro(2) formation. However, transfection of p3′-Δde greatly reduced expression in circScro(2), suggesting that both “d” and “e” regions are critical for circScro(2) production. Each bar represents mean ± SD (*n* = 3). **P < 0.05, ***P < 0.001, ****P < 0.0001 compared with pS-ICS-FF. C) Schematic of the hairpin loop involved in the biogenesis of circScro(2). The dashed arrow indicates back-splicing. Distinct regions within ICS are indicated by a–e. SS, splicing site.

To further investigate if there is a minimal or core region within the ICS for promoting circScro(2) production, the 105-nt ICS located in intron-2 (S-ICS2) was divided equally into five 21-nucleotide (nt)-long regions labeled from “a” to “e” (Fig. 5A and C). These regions were serially deleted in the 5′→3′ direction and designated as p5′-Δa, p5′-Δab, p5′-Δabc, and p5′-Δabcd, respectively (blue bars in Fig. 5A). Each of these constructs was tested in S2 cells for circScro(2) production. Expression levels of circScro(2) with p5′-Δa, p5′-Δab, and p5′-Δabc were slightly higher or comparable with FF control, whereas those with p5′-Δabcd were at the basal level as the negative control (pΔabcde) (blue bars, Fig. 5B). These results inform that the “d” region is critical for the hairpin formation. We also reasoned that higher yields of p5′-Δa and p5′-Δab might be because the deletion of these regions brought the ICS closer to E2.

Next, the serial deletions were made in the 3′→5′ direction (red bars in Fig. 5A). All of these constructs produced basal or slightly higher than basal levels of circScro(2) expression (red bars in Fig. 5B). We were surprised to see the lack of activity by p3′-Δe missing only “e” region because it includes the “d” region that we expected to be essential from the p5′-Δabcd result. Hence, we propose that the sequence spanning both “d” and “e” regions serves as a core ICS motif. The “d–e” region is the most distal part of the stem, positioned away from the loop region (Fig. 5C). It leads us to speculate that the “d–e” region might promote hairpin formation and/or interact with *trans*-factor(s) that play a role in hairpin formation or circularization. To see whether the core ICS is involved in hairpin structure, we aligned these sequences using EMBOSS to find inverted repeats needed for stem-loops in nucleotide sequences. However, except circScro(2) and circScro(3,4), none of the particular nucleotides needed for significant hairpin structure formation was found for other *scro* circRNAs.

### In vivo role of ICS for the production of *scro* circRNA

**Fig. 6.**
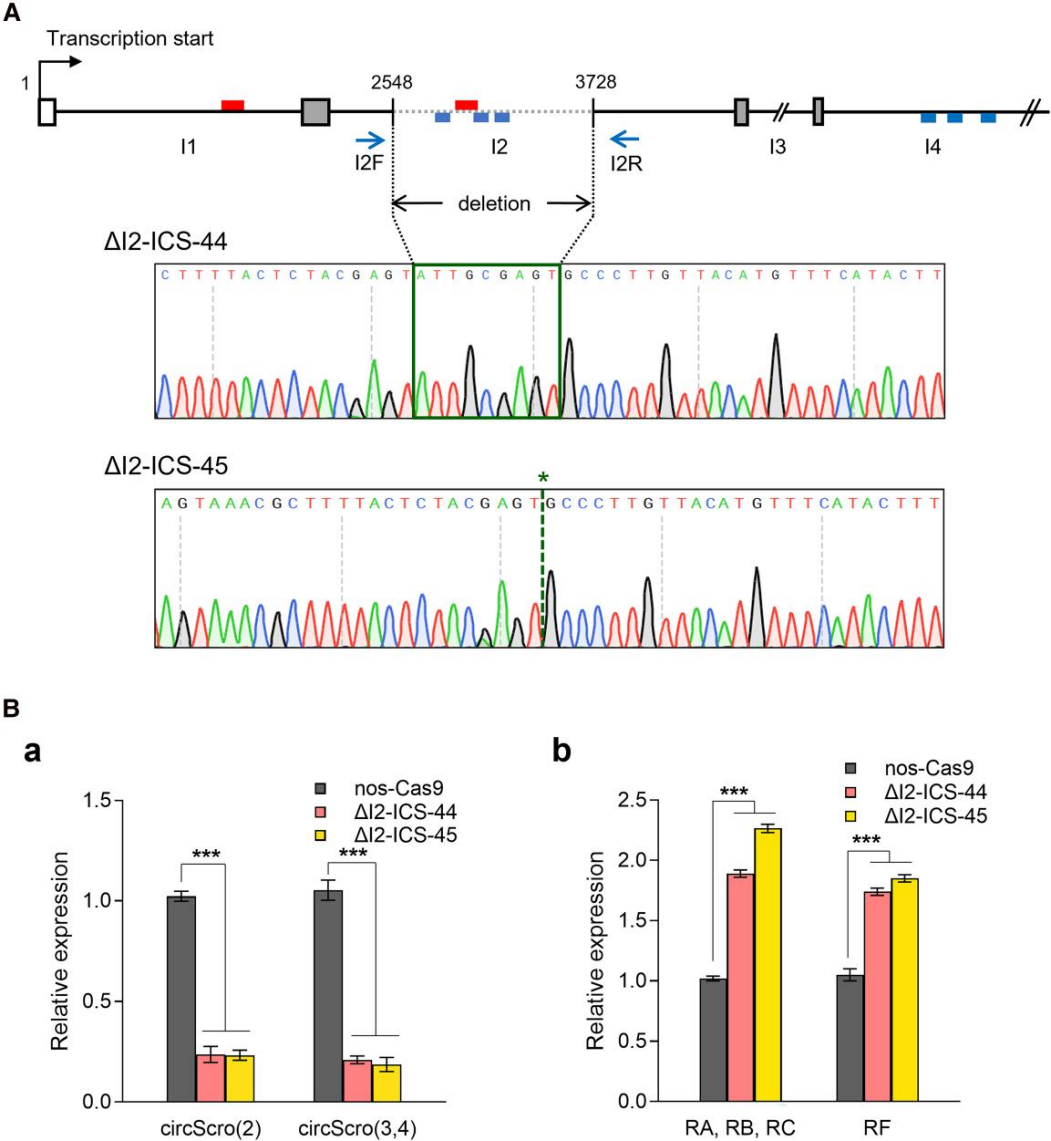
Analysis of the role of ICS in vivo. A) Schematic diagram showing the genomic deletion directed by gRNAs. The gRNAs were designed to remove the 1180-bp region containing ICS within the intron-2 (I2). The red boxes represent ICS for circScro(2), and the blue boxes represent candidate ICS regions for circScro(3,4). PCR using I2F and I2R primers was performed to determine the deleted region. The sequencing data show genomic lesions in both deletion lines. In *ΔI2-ICS-44*, 9 extra nucleotides within the green box are likely to be generated by an imprecise nonhomologous end-joining (NHEJ) mechanism during the double-strand repair. In *ΔI2-ICS-45*, the green asterisk indicates the gRNA-mediated cleavage site, displaying the precise deletion of the target region during the NHEJ process. Ba) The expression levels of circRNAs from the 2 homozygous deletion lines. Not only circScro(2) but circScro(3,4) levels were dramatically reduced when compared with those of the control. E2-2F and E2R2 primers were used for the detection of circScro(2), and E3-4F and E3-4R primers for circScro(3,4). Bars represent mean ± SD (*n* = 3). ***P < 0.001 compared with *nos-Cas9* control. Bb) The expression levels of linear *scro* transcript isoforms in the 2 homozygous deletion lines, *ΔI2-ICS-44* and *ΔI2-ICS-45*. In contrast to the Ba) result, the linRNA levels increased about 2-fold compared with those of the control. E1F2 and E2R2 primers were used for the detection of linear transcripts containing E2 (*RA*, *RB*, and *RC*) and E1-3F and E3R primers for *RF* transcript. Bars represent mean ± SD (*n* = 3). ***P < 0.001 compared with *nos-Cas9* control.

To further demonstrate the role of the identified S-ICS for circScro(2) expression in vivo, the CRISPR/Cas9-mediated genome editing tool was employed to remove an 1180-bp intronic region containing the S-ICS2. Using PCR-based screening, we identified and established 2 independent lines, Δ*I2-ICS-44* and *−*45 (Supplementary Fig. 3). The former carries the target region which is deleted but 9 ectopic nucleotides are inserted perhaps due to imprecise DNA repair, whereas the latter contains genomic lesion without an additional indel mutation (Fig. 6A). In contrast to early lethality of the *scro*-null mutants (Yoo *et al*. 2020), the new mutant flies showed no recognizable defects in their development and fertility. The survival rates of the deletion lines were also similar to that of *nos-Cas9* control (Supplementary Fig. 4). Remarkably, the expression levels of circScro(2) reduced by about 80% in the adult head of both mutant lines when compared with those in *nos-Cas9* control (Fig. 6Ba).

The deleted region contains not only the S-ICS2 essential for circScro(2) but also another potential ICS for circScro(3,4) production (Fig. 6A; Supplementary Fig. 2B), thus expecting to affect circScro(3,4) production. Indeed, circScro(3,4) levels showed a significant reduction as circScro(2) did in the mutants (Fig. 6Ba). These data together strongly support that ICSs are important for the in vivo production of both circScro(2) and circScro(3,4). Despite the significant reduction of circRNA levels, since the mutants look quite normal, either low levels of circScro(2) and circScro(3,4) are still sufficient for functioning or they are dispensable for larval and adult development. Complete elimination of these circRNAs, if possible, would address their in vivo roles more clearly.

We also like to note that expression levels of 2 types of linRNAs, one type including E2 (*RA*, *RB*, and *RC*) and the other E3 (*RF*), were about 2-fold greater in Δ*I2-ICS-44* and *-45* mutants than in *nos-Cas9* control (Fig. 6Bb). A likely possibility is that conventional splicing for the linRNAs and back-splicing for the circRNA compete for the same splicing machinery.

### Exons do not play a role in circRNA biogenesis

**Fig. 7.**
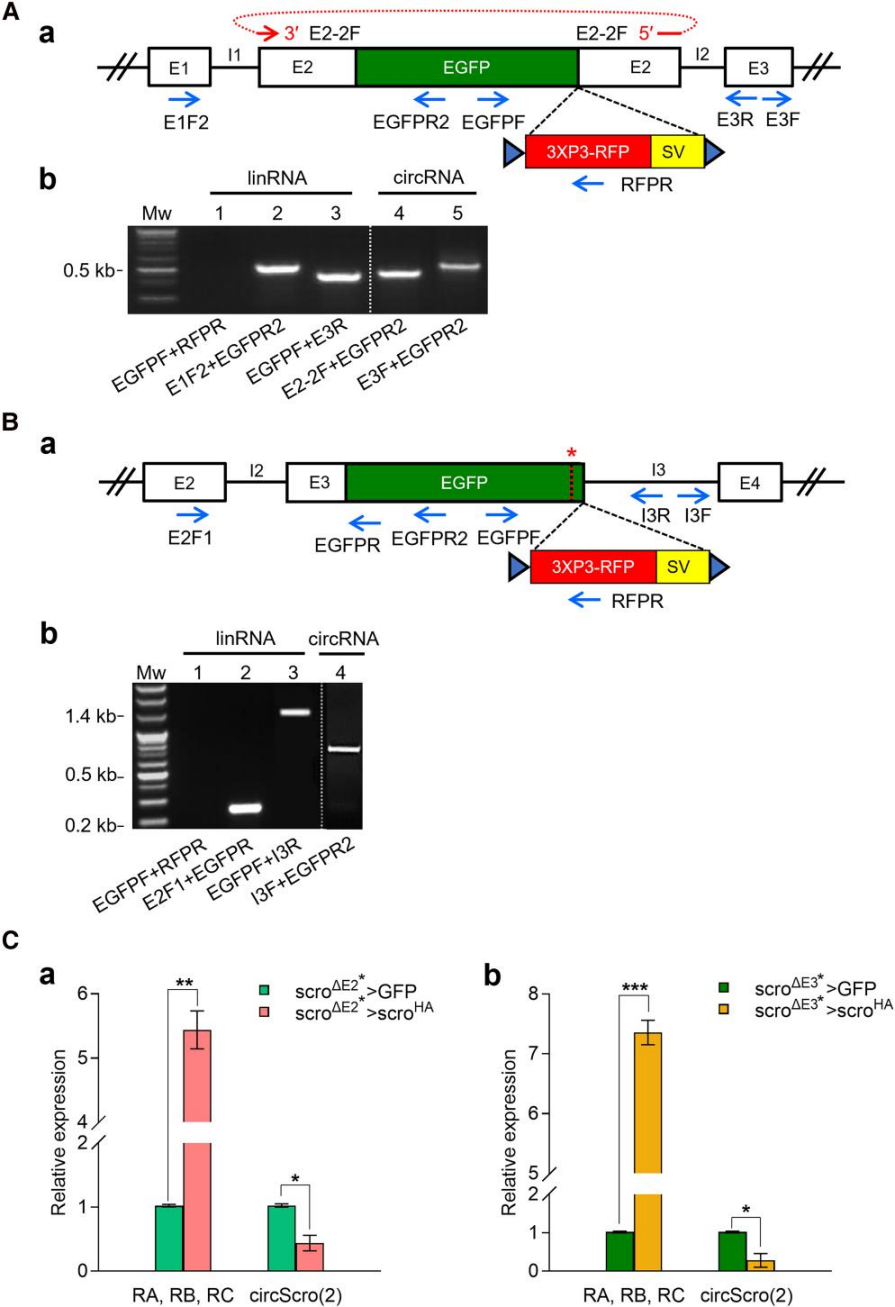
Analysis of circular transcripts from *scro* knock-in mutants. A) Genomic organization of *scro*^ΔE2−EGFP*^ knock-in mutant and RT-PCR results. Aa) The RFP marker and SV40 termination signal were removed from *scro*^*ΔE2−EGFP*^ using the Cre/loxP recombination system. The blue triangles represent the loxP sequence, and the red loop arrow above the diagram represents the E2-2F exon junction primer. Ab) cDNA was synthesized using oligo d(T) for linRNAs and hexamer for circRNAs. RT-PCR was performed using the primers indicated under each lane. The lack of PCR product in lane 1 verifies the removal of the RFP marker and SV40 terminator. The linRNAs carrying EGFP are produced normally (lanes 2 and 3). Note that circRNAs carrying E2/EGFP hybrid exon are also formed [lane 4, circScro(2/EGFP); lane 5, circScro(2/EGFP,3)]. See also the sequence data in Supplementary Fig. 5A and B. B) Genomic organization of *scro*^ΔE3−EGFP*^ knock-in mutant Ba) and RT-PCR results Bb). After the removal of the RFP marker and SV40 termination signal from *scro*^*ΔE3−EGFP*^, RT-PCR was performed. In agarose gel, the elimination of the RFP marker and SV40 terminator was confirmed in lane 1. Analysis of linear transcripts showed that the splicing of intron-2 occurs normally (lane 2), whereas intron-3 is not spliced out (lane 3). The circRNA containing E3/EGFP, the truncated I3, and E4 is confirmed by using I3F and EGFPR2 primers (lane 4; see also Supplementary Fig. 5C for the sequence data). Such an aberrant circRNA is most likely caused by the lack of the 5′ splice site in the I3 (red asterisk above the diagram). C) The expression levels of linear and circRNAs in response to transgenic overexpression of *scro* linRNA. Females of either *UAS-mCD8GFP*/*UAS-mCD8GFP*;; *scro*^ΔE2−Gal4*^/*TM6B* Ca) or *UAS-mCD8GFP*/*UAS-mCD8GFP*;; *scro*^ΔE3−Gal4*^/*TM6B* Cb) were crossed to *UAS-scro*^*HA*^ homozygote males, and then F1 progeny lacking *Tb* marker were selected for RNA purification from their heads. RT-qPCR was performed by using the E1F2/E2R2 primer set for linRNAs and the E2-2F/E2R2 primer set for circScro(2). The expression of linRNAs increased more than 5-fold, while circScro(2) levels reduced by 2- to 3-fold compared with the corresponding controls carrying *UAS-mCD8GFP* and either *scro*^ΔE2−Gal4*^/*TM6B* or *scro*^ΔE3−Gal4*^/*TM6B*, respectively. Bars represent mean ± SD (*n* = 3). *P < 0.05, **P < 0.01, ***P < 0.001 compared with the controls.

In addition to the ICSs, we wondered if there is any exonic contribution to circRNA production. To address this, we utilized previously generated *scro* knock-in mutants: *scro*^*ΔE2−EGFP*^, *scro*^*ΔE3−EGFP*^, *scro*^*ΔE2−Gal4*^, and *scro*^*ΔE3−Gal4*^ (Yoo *et al*. 2020). *scro*^*ΔE2−EGFP*^ was made by replacing most E2 with a cassette including EGFP, 3XP3-RFP marker, and SV40 terminator (Fig. 7Aa). For *scro*^*ΔE3−EGFP*^, the E3 and part of the flanking intron-3 were replaced with the same cassette (Fig. 7Ba). *scro*^*ΔE2−Gal4*^ and *scro*^*ΔE3−Gal4*^ (Gal4 knock-in) are the same as EGFP knock-in, except for the Gal4 coding sequence in place of EGFP. Since the SV40 terminator blocks the process of transcription, we first removed SV40 and 3XP3-RFP by using the Cre/lox recombination system (Fig. 7Aa and Ba). These excision lines were differentially designated from the original ones by adding an asterisk to the end as follows: *scro*^ΔE2−EGFP*^ or *scro*^ΔE2−Gal4*^ and *scro*^ΔE3−EGFP*^ or *scro*^ΔE3−Gal4*^.

We tested if the hybrid E2 (E2/EGFP) exon influences circRNA and/or linRNA production. Since the knock-in mutants were homozygous lethal during late embryogenic and early larval stages, heterozygous flies were used to detect circScro(2/EGFP). The results showed that splicing of intron-1 and intron-2 occurred normally to produce linRNA containing E2/EGFP exon (Fig. 7Ab, lanes 2 and 3, respectively). We also found single-exonic circRNA carrying E2/EGFP exon (Fig. 7Ab, lane 4) and confirmed it by sequencing (Supplementary Fig. 5A). In addition, bi-exonic circScro(2/EGFP,3) was also detected (Fig. 7Ab, lane 5; Supplementary Fig. 5B). These results are consistent with the previous finding that the endogenous exon sequence is unlikely to contribute to the circularization process (Kramer *et al*. 2015).

### Splicing junction mutation can cause aberrant biogenesis of circRNA

As mentioned earlier, our painstaking efforts did not detect any *scro* circRNAs containing intronic sequences in wild type (Fig. 2). This is likely because the introns are rapidly spliced out during the biogenesis of the circRNAs. If so, it is possible that mutations removing a splicing junction site cause the production of unusual circRNAs containing intronic sequences. To test this in vivo, we took advantage of the *scro*^ΔE3−EGFP*^ line in which EGFP knock-in deletes the 5′ portion of intron-3 (I3) including the 5′ splicing site (Fig. 7Ba; Yoo *et al*. 2020). Accordingly, we anticipated the lack of splicing of the intron-3. RT-PCR results using oligo d(T)-primed cDNA template confirmed that the intron-2 (I2) is spliced out, but the I3 remains in the linear transcript derived from *scro*^ΔE3−EGFP*^ (Fig. 7Bb, lanes 2 and 3, respectively). This result implies that the truncated I3 (tI3) is part of a new exon combining E3/EGFP and E4 (E3/EGFP-tI3-E4). To test if this new exon can generate a circRNA, RT-PCR was performed using the hexamer-primed cDNA template and a divergent primer pair, EGFPR2 and I3F. As a result, we obtained the 722-bp fragment and verified it by sequencing (Fig. 7Bb lane 4; Supplementary Fig. 5C). Although we were able to detect this circRNA by end-point PCR, qPCR results showed that its levels were quite low (data not shown), when compared to the circScro(3,4) that is one of the 2 most abundant forms (Fig. 3A). Nevertheless, our data suggest that splicing junction mutations can cause the production of aberrant circRNAs, let alone aberrant linRNAs.

### Overexpressed Scro alters the transcript levels of endogenous *scro*

We also wanted to see if overexpressed Scro alters the production levels of *scro*'s linRNA and/or circRNAs. To test this, the *scro*^ΔE2−Gal4*^ knock-in line was crossed to *UAS-scro*^*HA*^. Since the *UAS*-*scro*^*HA*^ transgene does not contain E1 and any of the introns, circRNAs are unlikely to be produced from this construct. We then measured levels of linRNAs and circRNAs derived “exclusively” from the endogenous *scro* gene. For the linRNAs, a set of E1F2 and E2R2 primers was used as the *UAS-scro*^*HA*^ transgene-derived mRNA lacks the E1; for the circScro(2), E2-2F and E2R2 primers were employed since the E2R2 binding site is absent in the Gal4 transgene (Supplementary Fig. 6). Interestingly, we found that the linRNA levels increased by 5–6-fold (Fig. 7Ca), inferring an auto-regulatory mechanism in which Scro enhances the expression of its gene. On the other hand, circScro(2) levels decreased by 2–3-fold than the control (Fig. 7Ca). Progeny from a crossing of *UAS-scro*^*HA*^ to *scro*^ΔE3−Gal4*^ showed similar results (Fig. 7Cb). These data are somewhat consistent with the foregoing ones (Fig. 6B) in which lower levels of circRNA are concomitant with higher levels of linRNA. These data imply that Scro somehow modulates the ratio between linear and circRNA forms by a novel mechanism. We speculate that the balanced production of linRNA and circRNA is subjected to change in response to various cellular environments that alter the production of one or the other RNA form.

## Discussion

The circRNAs are first thought to have arisen from aberrant splicing of pre-mRNAs (Cocquerelle *et al*. 1992, 1993). However, it is now widely accepted that circRNAs are one of the major classes of noncoding RNAs, and their biogenesis is delicately regulated (Salzman *et al*. 2012; Kristensen *et al*. 2019). In *Drosophila*, more than 2,500 circRNAs have been annotated (Ashwal-Fluss *et al*. 2014; Westholm *et al*. 2014). However, the biogenesis of most circRNAs is little understood. In this study, we undertook comprehensive molecular analyses of the *scro*-derived circRNAs to gain insights into the biogenesis mechanism of *scro* circRNAs in both in vivo and cell culture systems as well as their possible roles.

We identified 12 circRNAs including the one previously reported (Westholm *et al*. 2014), and all of them are either single or multiexonic circRNAs. The presence of multiexonic ones indicates that interexonic introns are processed out after or during circularization, supporting that conventional splicing for linRNAs, and back-splicing events might utilize the same or similar splicing mechanism(s). Interestingly, 6 circRNAs start with E2 and 4 with E3; since E3 is the second exon of *RF* transcript, our results are consistent with previous reports showing the second exon–biased circularization and cotranscriptional splicing for circRNA formation (Ashwal-Fluss *et al*. 2014; Westholm *et al*. 2014; Zhang *et al*. 2014). We did not find circRNAs containing E1 or E7. It is consistent with previous findings showing that the first or last exon of a gene is rarely made into circRNAs because they are flanked by only one intron (Salzman *et al*. 2012; Lasda and Parker 2014; Westholm *et al*. 2014; Gruner *et al*. 2016). We also did not find circRNAs containing E6, although this exon is flanked by the 2 largest introns.

Back-splicing mediated circularization is expected to involve both *cis*- and *trans*-factors (Kramer *et al*. 2015; Kristensen *et al*. 2019). As for the *cis*-factors, we showed that a hairpin structure involving 105-bp ICS in the E2-flanking introns is crucial for circScro(2) formation both in cultured cells and in vivo. In addition to the ICS, the regions immediately upstream and downstream of the E2 are likely to be important (Fig. 4). Since these regions contain splicing junction sites, it seems that they are required for the basal level of back-splicing, whereas ICS enhances this event significantly. Another ICS pair located in intron-2 and intron-4 is likely to play a role in enhancing circScro(3,4) production. Aligning sequences of introns flanking other exons did not reveal candidate ICSs, raising the possibility of alternative mechanisms to produce other circRNAs.

Another notable feature of the ICSs is that the distance between the ICS and the splicing junction site influences the circularization efficiency. As shown in Fig. 4, the ICS closer to the splicing junction site increased circRNA production levels perhaps by enhancing the rates of hairpin formation. ICSs mediate the formation of a hairpin structure that divides into 2 distinct parts, a loop and a stem. Interestingly, the most distal stem region from the loop (d and e in Fig. 5A) is critical for circRNA production, although the degree of base-pairing is greater in the proximal a and b regions (Supplementary Fig. 2A). Perhaps, the distal region is important for initiating the ICS pairing and/or for binding a *trans*-acting factor that is involved in the circularization process.

The back-splicing also needs *trans*-factors, and currently, a few RBPs are found to play roles in the biogenesis of *Drosophila* circRNAs (Kramer *et al*. 2015; Pamudurti *et al*. 2022). For example, splicing factors, hnRNP (heterogenous nuclear ribonucleoprotein), and serine-arginine proteins combinatorially regulate the circRNA levels derived from the *Drosophila laccase2* and *Plexin A,* but not those from *mbl* (Kramer *et al*. 2015). These studies suggest a mechanism in which sequence-specific RBPs regulate circRNA biogenesis in a gene-specific manner. Interestingly, transgenic *scro* overexpression resulted in a significant reduction in circRNA production but an increase in linRNAs from the endogenous *scro* locus (Fig. 7C). Since Scro functions as a homeodomain transcription factor, it is unlikely that overexpressed Scro suppresses circularization directly (Nair *et al*. 2020; Yoo *et al*. 2020; Konstantinides *et al*. 2022). It could be an indirect effect through the expression of a Scro-target gene that encodes either an RBP or an RBP-regulating factor, which in turn controls the ratio of linear RNA to circRNA. In this case, identifying RBPs binding to the *scro* ICSs and flanking intron sequences will shed light on the biogenesis mechanism of *scro* circRNAs.

The molecular functions of circRNA are beginning to be understood (reviewed by Li *et al*. 2018; Kristensen *et al*. 2019). Relatively well-known roles include the regulation of gene expression via their binding to complementary microRNAs (miRNA sponge) or RBPs (protein sponge). Searching for the miRBase (Kozomara and Griffiths-Jones 2011) revealed miR-958-3p and miR-994-5p that show a substantial degree of complementarity to *scro* E2 and E4, respectively (Supplementary Fig. 7). While the function and biological process of miR-994 are unknown, miR-958 was reported to regulate Hedgehog signaling-mediated patterning of the wing imaginal disc (He *et al*. 2022). It will be interesting to investigate if *scro* circRNAs play a role as a sponge of these miRNAs, thereby affecting expression levels of these miRNA-target genes. A few exonic circRNAs containing a short open-reading frame are translated to produce truncated proteins which may or may not be functional (e.g. Pamudurti *et al*. 2017). According to the sequence data, certain *scro* circRNAs contain short open-reading frames, raising the possibility that *scro* circRNAs produce truncated peptides. The roles of these peptides, if they are truly produced, will be an interesting subject of future study.

Biological roles of circRNAs may be associated with aging. We have shown that the levels of circScro(2) progressively escalated in aged fly heads. Likewise, the aging-dependent accumulation of circRNAs in fly heads is also observed for *mbl*, *CaMK-like*, *p120 catenin*, and *ankyrin 2* genes (Ashwal-Fluss *et al*. 2014; Westholm *et al*. 2014). Hence, circRNAs are considered a new indicator of the aging brain in *Drosophila* and mammals (Westholm *et al*. 2014; Gruner *et al*. 2016; Knupp and Miura 2018). Interestingly, aging *Drosophila* eyes show altered expression of splicing-related genes, which are causally related to an accumulation of circRNAs and visual senescence (Hall *et al*. 2017; Stegeman *et al*. 2018). Since *scro* transcripts are enriched in the optic lobe medulla in adult flies (Yoo *et al*. 2020), it will be interesting to resolve if accumulated *scro* circRNA in the aged visual nervous system is also due to altered splicing factors and whether it contributes to aging-associated decline of visual function. Interestingly, the level of circRNA derived from a *sulfateless* gene is positively associated with longevity (Weigelt *et al*. 2020). By comparison, *scro* circRNAs are unlikely to be involved in longevity, as our ICS deletion mutants, which carry only 20% of *scro* circRNAs, showed normal survival rates. However, whether the complete elimination of *scro* circRNA or overexpression of it alters the longevity needs to be tested.

## Supplementary Material

jkaf055_Supplementary_Data

## Data Availability

We affirm that all data necessary for confirming the conclusions of the article are present within the article, figures, and tables. Stocks are available upon request. Supplemental material available at G3 online.
