# Supplementary material for: Biogenesis of circular RNAs in vitro and in vivo from the Drosophila Nk2.1/scarecrow gene
Source: G3 (Bethesda). 2025 Mar 12;15(5):jkaf055. doi: 10.1093/g3journal/jkaf055 (PMC12060249; doi:10.1093/g3journal/jkaf055)
Supplement: jkaf055_Supplementary_Data [file jkaf055_supplementary_data.zip › Supplementary_Figure_Legends_G3-2025-405711.docx]

**Supplementary Figures**

**Supplementary Fig. 1. Sequence data showing exon junction of all circRNAs.**

The red asterisks (*) indicate the exon-exon joining site as a result of back-splicing.

**(A)** Head-to-tail splice junctions of circRNAs containing exon-2 are derived from single-exon circular form, circScro(2) **(Aa)**, bi-exonic circScro(2,3), circScro(2,4), and circScro(2,5) **(Ab-d)**, tri-exonic circScro(2,3,4) **(Ae)**, and tetra-exonic circScro(2,3,4,5) **(Af)**.

**(B)** Head-to-tail splice junctions of circRNAs containing exon-3 are derived from single-exon circular form, circScro(3) **(Ba)**, bi-exonic circScro(3,5) and circScro(3,4) **(Bb-c)**, and tri-exonic circScro(3,4,5) **(Bd)**.

**(C)** Head-to-tail splice junctions of circRNAs containing exon-4 are derived from single-exonic circScro(4) **(Ca)** and bi-exonic circScro(4,5) **(Cb)**.

**Supplementary Fig. 2. Identification of candidate intronic complementary sequences (ICSs) *in silico*.**

The numbers indicate positions relative to the transcription start site.

**(A)** Base-pairing region between intron-1 (I1) and intron-2 (I2) shows 105-bp ICS for circScro(2) (see also Figs. 4-6 for the results related to this ICS).

**(B)** Three candidate ICS regions between intron-2 (I2) and intron-4 (I4) for circScro(3,4) (blue boxes in Fig. 6A).

**(C)** Base-pairing region between intron-1 (I1) and intron-2 (I2) from the *laccase2* gene displays 206-bp ICS for *laccase2* circRNA (see the related experiments in Fig. 4Ac).

**Supplementary Fig. 3. Generation of the *scro* mutants lacking ICS.**

Crossing scheme of the injected nos-Cas9. Fly images were created on BioRender (https://www.biorender.com/).

**Supplementary Fig. 4. Survival rates of the ICS deletion lines.**

Approximately 100 adult flies were collected after hatching and transferred to five vials containing equal numbers of males and females. The flies were raised at 25°C and transferred into fresh food vials every 3 days. Surviving flies were counted daily for up to 50 days. The survival rates of both deletion mutants were not significantly different from that of the *nos-Cas9* control group, suggesting that there is no correlation between the levels of circScro and longevity (*n*=3).

**Supplementary Fig. 5. Verification of circRNA carrying E2/EGFP and E3/EGFP hybrid exons.**

**(Aa)** Schematic diagram of circScro(2/EGFP) generated from *scro^ΔE2-EGFP*^* knock-in mutant. **(Ab)** Sequencing data showing head-to-tail splice junction of E2 (red asterisk) and E2/EGFP junction site (blue asterisk). **(Ba)** Schematic diagram of circScro(2/EGFP,3) derived from *scro^ΔE2-EGFP*^* knock-in mutant. **(Bb)** Sequencing data showing head-to-tail splice junction of E2 and E3 (red asterisk) and E2/EGFP junction site (blue asterisk). **(Ca)** Schematic diagram of circScro(3/EGFP,tI3,4) derived from *scro^ΔE3-EGFP*^* knock-in mutant. **(Cb)** Sequencing data representing head-to-tail splice junction of E3 and E4 (red asterisk) and E3/EGFP junction site (blue asterisk). The *EcoR*I site was used for cloning to generate knock-in mutant flies (Yoo et al. 2020).

**Supplementary Fig. 6. PCR strategy for detecting endogenously expressed linRNA and circScro(2**) **in *scro*-overexpressing flies.**

PCR strategy related to Fig. 7Ca of detecting *scro* linRNA (using E1F2/E2R2 primers) and circScro(2) (using E2-2F/E2R2 primers) exclusively from the native exon. Due to the lack of an E2R2 binding site in the Gal4 knock-in exon **(A)** and the lack of E1 in the *UAS-scro* transgene **(C)**, both linRNA and circScro(2) can be generated only from the native E2 **(B)**.

**Supplementary Fig. 7. Candidate miRNAs targeted by *scro* circRNA.**

By using the miRBase web tool (<https://www.mirbase.org/>), we found candidate miRNAs that can bind to *scro* circRNA. **(A)** Complementarity between a region of *scro* E2 and miR-958-3p. **(B)** Complementarity between a region of *scro* E4 and miR-994-5p. Paired nucleotides are shown in bold-faced capitals.
