# Supplementary material for: Biogenesis of circular RNAs in vitro and in vivo from the Drosophila Nk2.1/scarecrow gene
Source: G3 (Bethesda). 2025 Mar 12;15(5):jkaf055. doi: 10.1093/g3journal/jkaf055 (PMC12060249; doi:10.1093/g3journal/jkaf055)
Supplement: jkaf055_Supplementary_Data [file jkaf055_supplementary_data.zip › Supplementary_Tables_G3-2025-405711.docx]

**Supplementary Table 1. List of primers used for cloning.**

| **Name** | **Sequence (5′→3′)** |
| --- | --- |
| scro-I1-NoICS-F | gcg**GGATCC**GTGTCAGTGAATTGATAAAAAATGG* |
| scro-I2-NoICS-R | cgc**GGTACC**GAACAGCATCTCACCGAATG |
| scro-I1-BF | gcg**GGATCC**GCCACGCCCTTTCTTACGCC |
| scro-I1-BR | cgc**GGATCC**AAAAAAACAAGAGAGGAAGCTATAGTC |
| scro-I2-KF | cgc**GGTACC**AACGAATCTAGTAAACGCTTTTAC |
| scro-I2-KR | cgc**GGTACC**CGATCGCGCAACTAACGCAC |
| laccase2-I1-BF | cgc**GGATCC**GGGCGGGTTTAGGGCGTG |
| laccase2-I1-BR | cgc**GGATCC**TACTAGATTCGTTGAAAAATAGGT |
| laccase2-I2-KF | cgc**GGTACC**TAGATTCGTTGAAAAGTATGTAAC |
| laccase2-I2-KR | cgc**GGTACC**GTGTGGGCGTGACCAAAT |
| scro-I2-5′-Δa-F | ATCAGACATATATGT---TATAGTCGAGTTCCC** |
| scro-I2-5′-Δa-R | GGGAACTCGACTATA---ACATATATGTCTGAT |
| scro-I2-5′-Δab-F | ATCAGACATATATGT---TCAGATACGCGTTAC |
| scro-I2-5′-Δab-R | GTAACGCGTATCTGA---ACATATATGTCTGAT |
| scro-I2-5′-Δabc-F | ATCAGACATATATGT---AGTAGAAATGCGAAC |
| scro-I2-5′-Δabc-R | GTTCGCATTTCTACT---ACATATATGTCTGAT |
| scro-I2-5′-Δabcd-F | ATCAGACATATATGT---TATCAAAATTTTTCT |
| scro-I2-5′-Δabcd-R | AGAAAAATTTTGATA---ACATATATGTCTGAT |
| scro-I2-3′-Δe-F | AATGCGAACGAGAAA---TAAAATAAGTTAATT |
| scro-I2-3′-Δe-R | AATTAACTTATTTTA---TTTCTCGTTCGCATT |
| scro-I2-3′-Δde-F | ACGCGTTACTCGGCT---TAAAATAAGTTAATT |
| scro-I2-3′-Δde-R | AATTAACTTATTTTA---AGCCGAGTAACGCGT |
| scro-I2-3′-Δcde-F | CGAGTTCCCCGACTA---TAAAATAAGTTAATT |
| scro-I2-3′-Δcde-R | AATTAACTTATTTTA---TAGTCGGGGAACTCG |
| scro-I2-3′-Δbcde-F | AACAATAGAGAACGC---TAAAATAAGTTAATT |
| scro-I2-3′-Δbcde-R | AATTAACTTATTTTA---GCGTTCTCTATTGTT |
| scro-I2-Δabcde-F | ATCAGACATATATGT---TAAAATAAGTTAATT |
| scro-I2-Δabcde-R | AATTAACTTATTTTA---ACATATATGTCTGAT |
| ΔI2-ICS-gRNA1-S | CTTCGTTACTCTACGAGTATTATGA*** |
| ΔI2-ICS-gRNA1-AS | AAACTCATAATACTCGTAGAGTAAC |
| ΔI2-ICS-gRNA2-S | CTTCGCATGTAACAAGGGCACAAAT |
| ΔI2-ICS-gRNA2-AS | AAACATTTGTGCCCTTGTTACATGC |

* Bold letters represent restriction enzyme sites, and the small cases are flanking sequences.

** The dashed line represents the deleted region.

*** In gRNA, single-stranded overhangs are underlined.

**Supplementary Table 2. List of primers used for PCR.**

| **Name*** | **Sequence (5′→3′)** |
| --- | --- |
| E1F2 | GTACGTCCTGTTACGTCC |
| E2F1 | CTGGAACTGAACGGAAAT |
| E2F2 | CGGCCTTGCTTACACAACAAG |
| E2R | GTGGTGGTGATGTGCAACCGC |
| E2R2 | CGCGGCTGCTGTAGATAC |
| E3F | CTGGCTGGTCACTACACTGAC |
| E3R | AATAGGTCTGTACACCTGGGC |
| E3R2 | GGTTATCGGTAGGTCCGC |
| E4R2 | CTAAAGGAAACTGTAGCG |
| E4R3 | CTCTTCGCTTTCTTCTTTGTG |
| E5F2 | CGAGAACACCTGGCTAGT |
| EGFPF | AACCACTACCTGAGCACCCAG |
| EGFPR | GCTTGCCGTAGGTGGCATCG |
| EGFPR2 | CCCTTCAGCTCGATGCGGTTC |
| RFPR | GGGCACCGTGAACAACCA |
| I2F | CGGGTGGTTTATGGACGC |
| I2R | CGTTTATTTCAGAACGTAGCC |
| I3F | GCGCCCATTTAGCCTCTCAG |
| I3R | CCATCGTACTATCGTTAAAGCG |
| rp49F | CACCAGGAACTTCTTGAATCC |
| rp49R | AGATCGTGAAGAAGCGCACCA |
| actin5CF | CCAGAGACACCAAACCGAAAG |
| actin5CR | AGTTGCTGCTCTGGTTGTCGA |

* F stands for forward and R for reverse orientation.

**Supplementary Table 3. List of exon junction primers used for PCR.**

| **Name** | **Sequence (5′→3′)** |
| --- | --- |
| E1-3F | GGTGCATAAAAAGAAATAAAAATC* |
| E2-2F | CACCATCTCCGTTTCGCG |
| E2-2R | GGTAGTAGGCTTTTATCGCG |
| E2-3F | ACCATCTCCGTTTCGATC |
| E2-3R | GTAGTAGGCTTTTATCGTTGC |
| E2-4F | CCATCTCCGTTTCGTTTC |
| E2-4R | GTAGGCTTTTATCGCTGG |
| E2-5F | ACCATCTCCGTTTCGGTG |
| E2-5R | AGGCTTTTATCGCTGCGT |
| E3-3F | ACCCAAGATTTGCAAATC |
| E3-2R | GCTGCTACTAGAGTTTGATCG |
| E3-4F | CCCAAGATTTGCAATTTC |
| E3-4R | CTAGAGTTTGATCTGGGC |
| E3-5F | ACCCAAGATTTGCAAGTG |
| E3-5R | ACTAGAGTTTGATCTGCG |
| E4-4F | ACGCAAGCCCAGTTTCAC |
| E4-5F | TTTACGCAAGCCCAGGTG |
| E4-5R | CATTAGGCGTGAAACTGC |

*Each exon junction primer was designed to prevent amplification from linRNAs. A short sequence underlined at the 3' end is complementary to the conjoined exon only in the circRNA context. This region would mismatch linRNA exons, thereby disallowing it to make a PCR product.
