# Supplementary figures and images for: Biogenesis of circular RNAs in vitro and in vivo from the Drosophila Nk2.1/scarecrow gene
Source: G3 (Bethesda). 2025 Mar 12;15(5):jkaf055. doi: 10.1093/g3journal/jkaf055 (PMC12060249; doi:10.1093/g3journal/jkaf055)

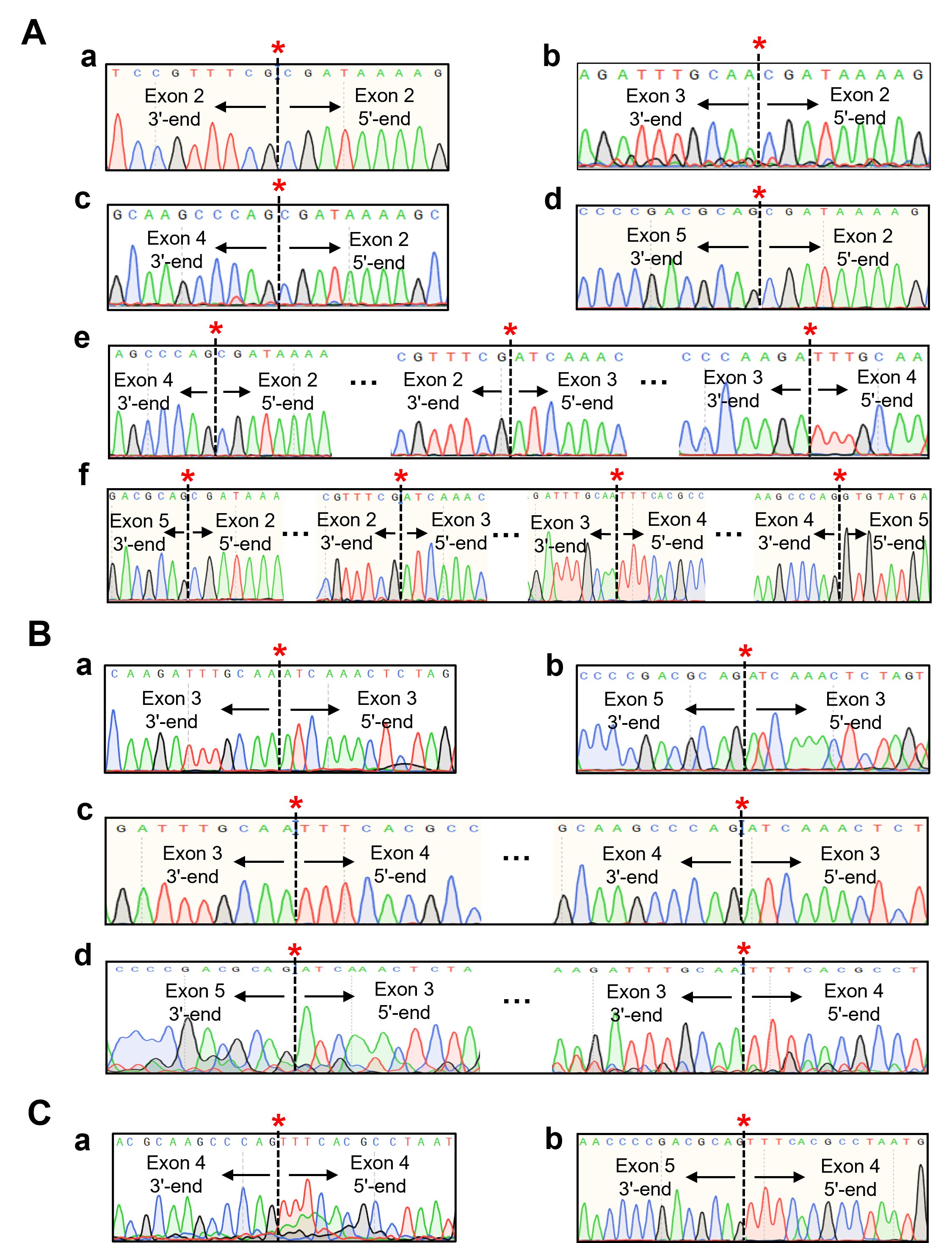

Supplement: jkaf055_Supplementary_Data [file jkaf055_supplementary_data.zip › Supplementary_Figure_1_G3-2025-405711.tif]

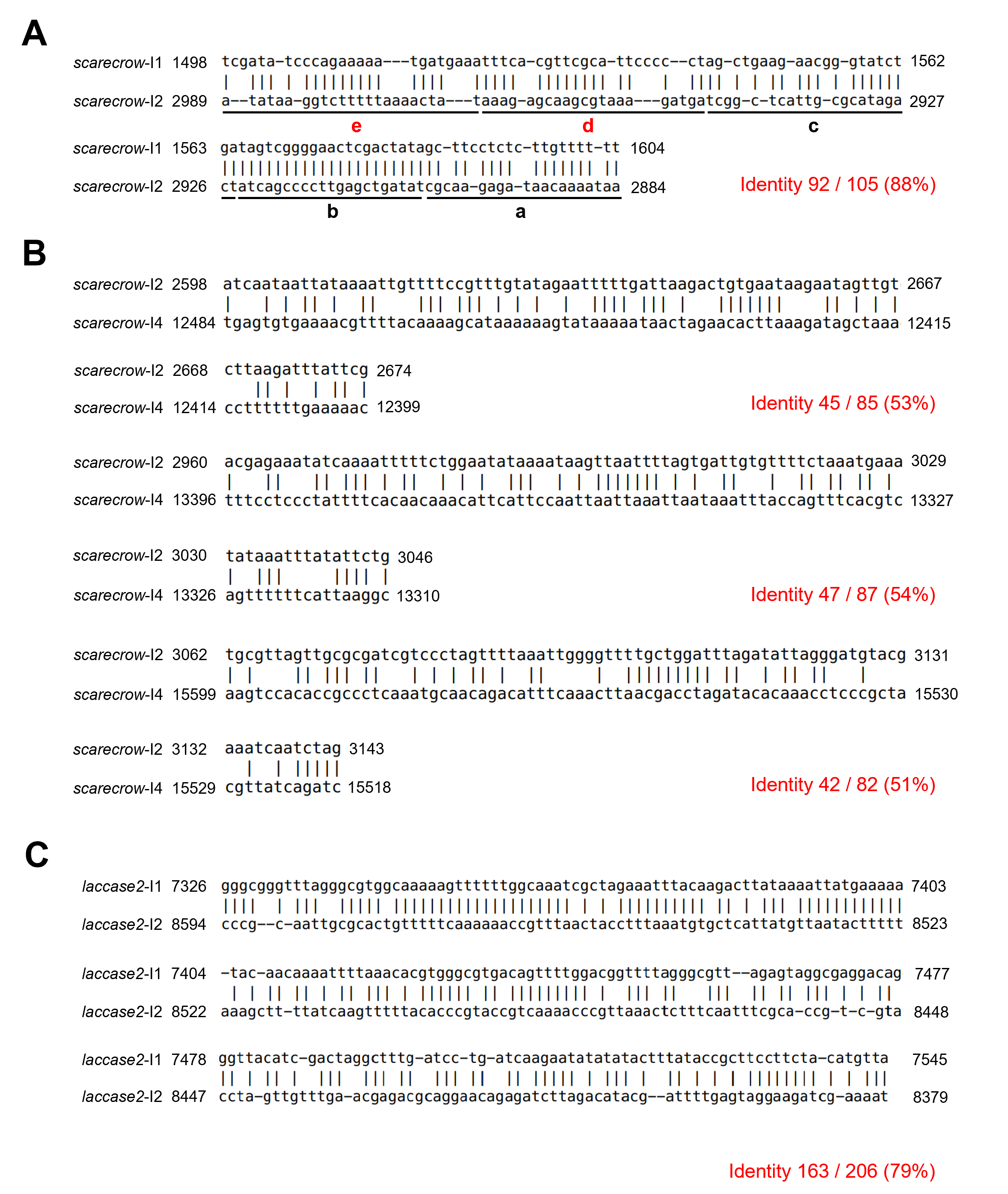

Supplement: jkaf055_Supplementary_Data [file jkaf055_supplementary_data.zip › Supplementary_Figure_2_G3-2025-405711.tif]

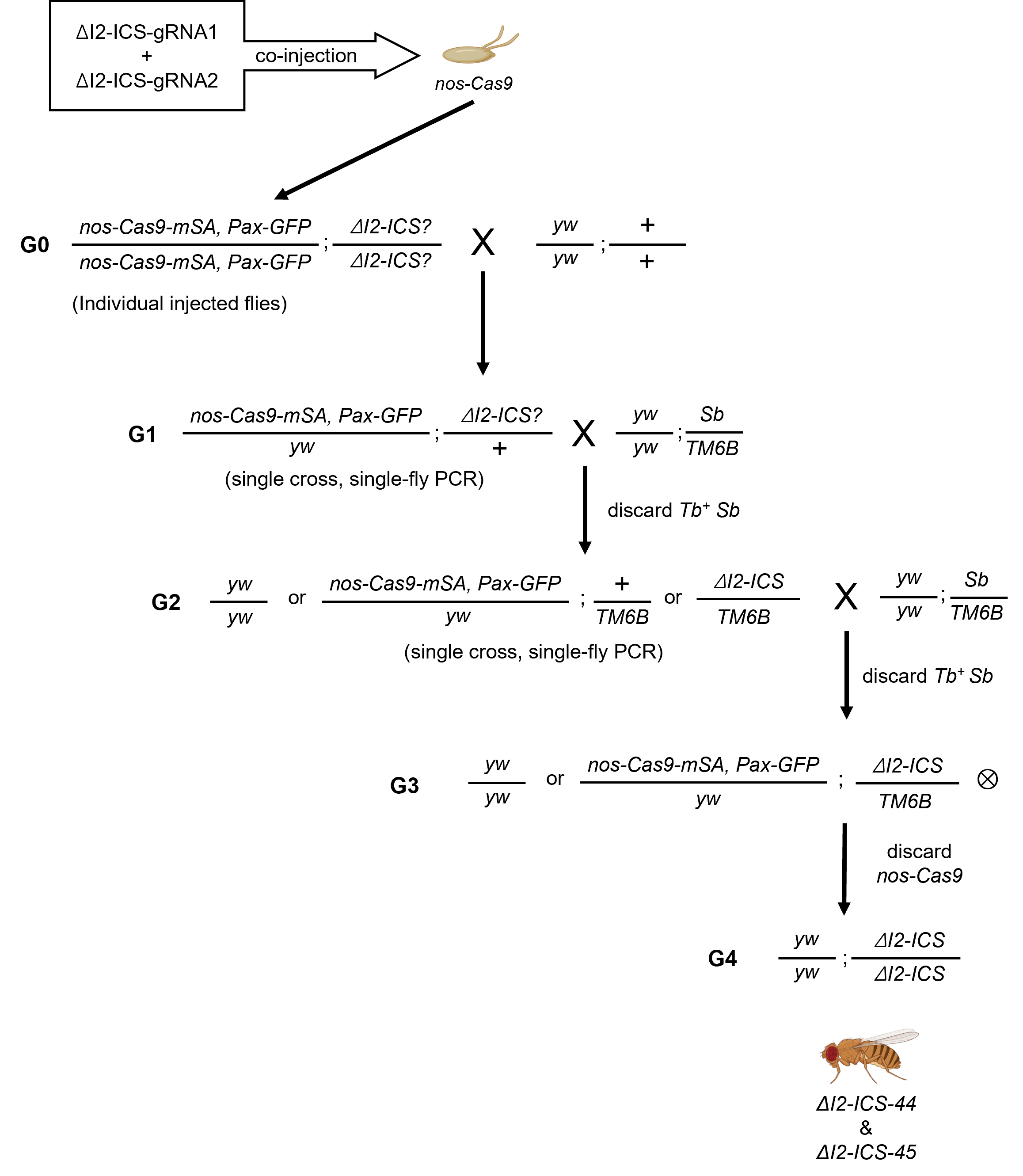

Supplement: jkaf055_Supplementary_Data [file jkaf055_supplementary_data.zip › Supplementary_Figure_3_G3-2025-405711.tif]

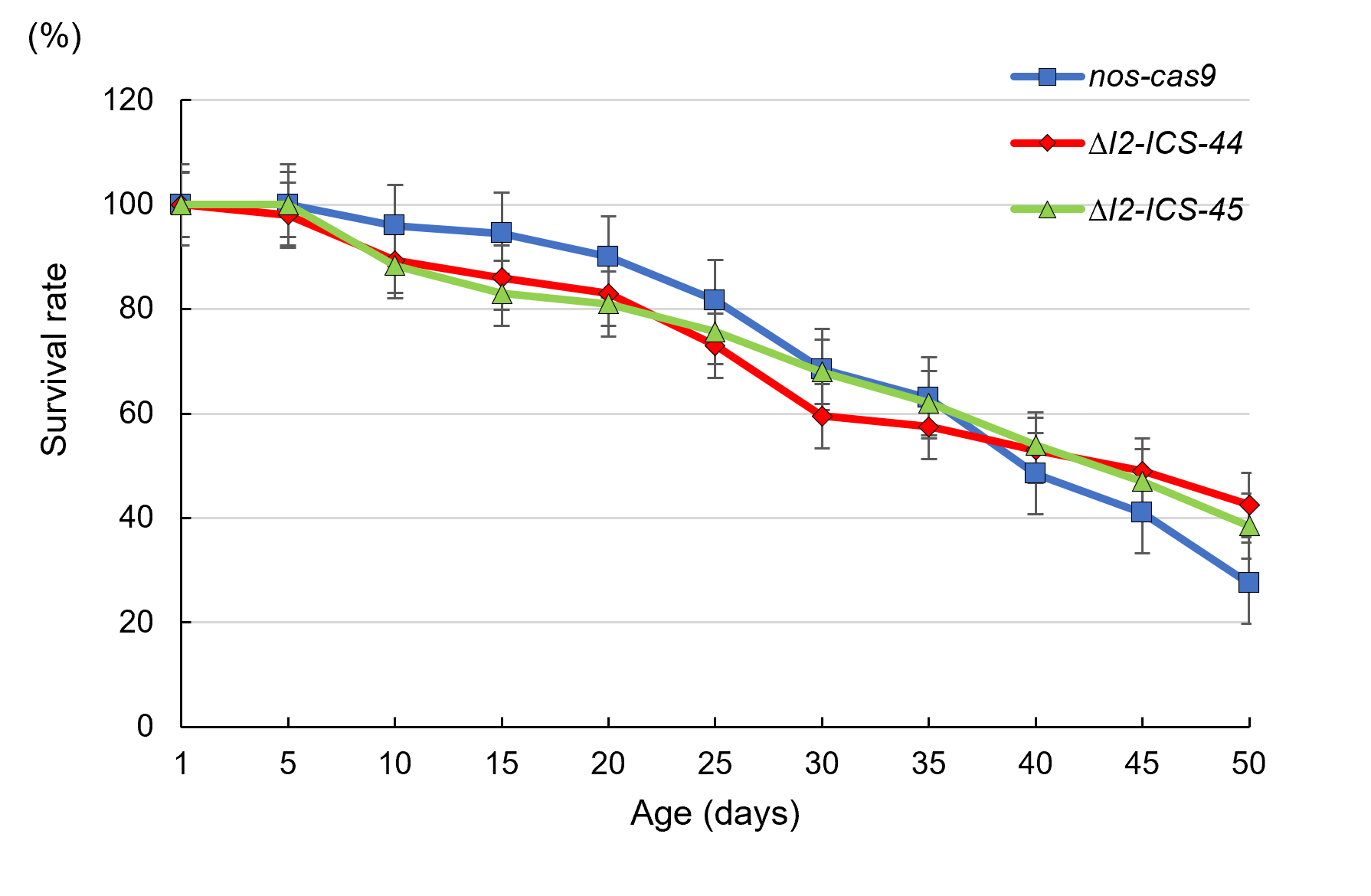

Supplement: jkaf055_Supplementary_Data [file jkaf055_supplementary_data.zip › Supplementary_Figure_4_G3-2025-405711.tif]

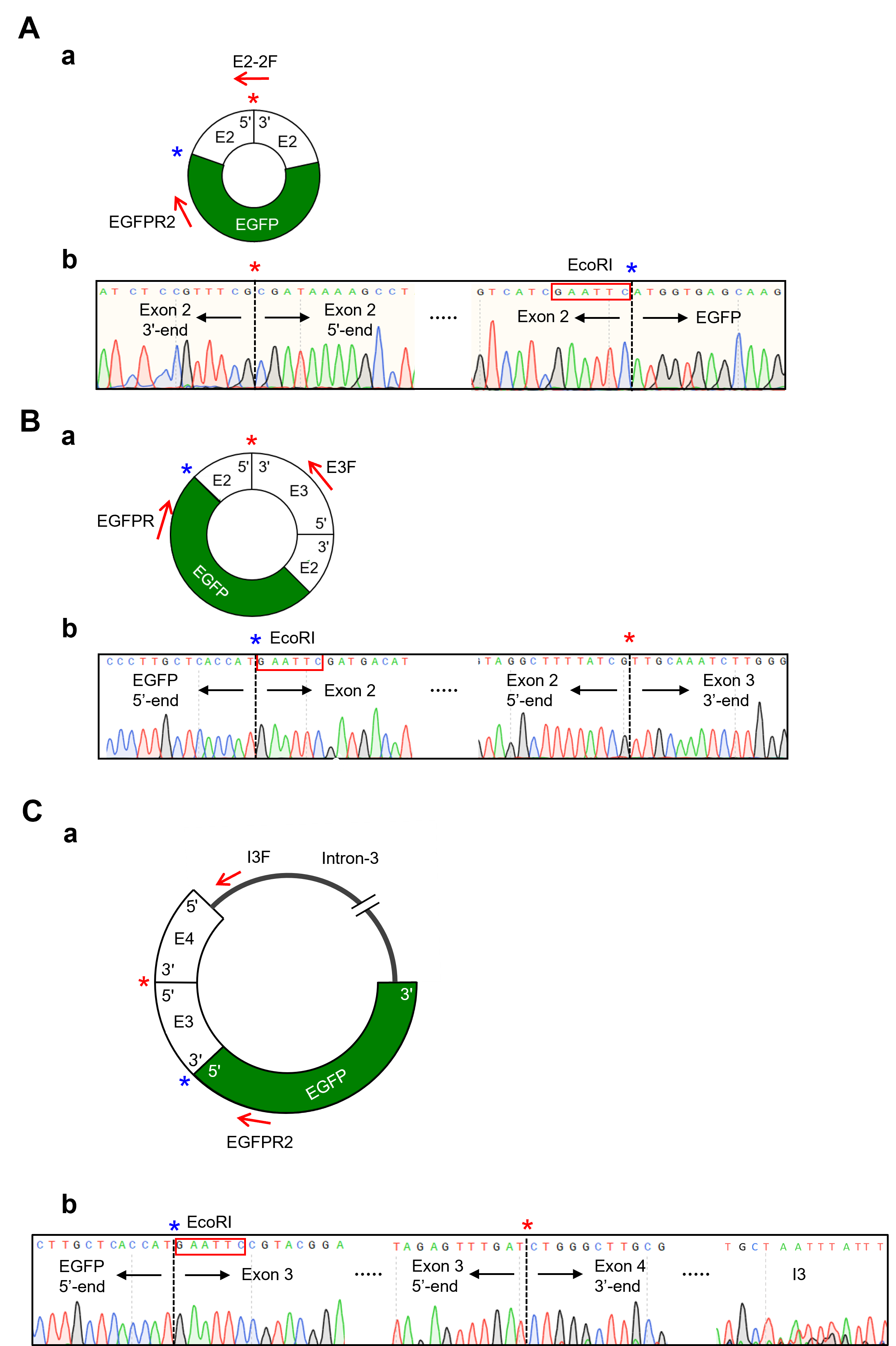

Supplement: jkaf055_Supplementary_Data [file jkaf055_supplementary_data.zip › Supplementary_Figure_5_G3-2025-405711.tif]

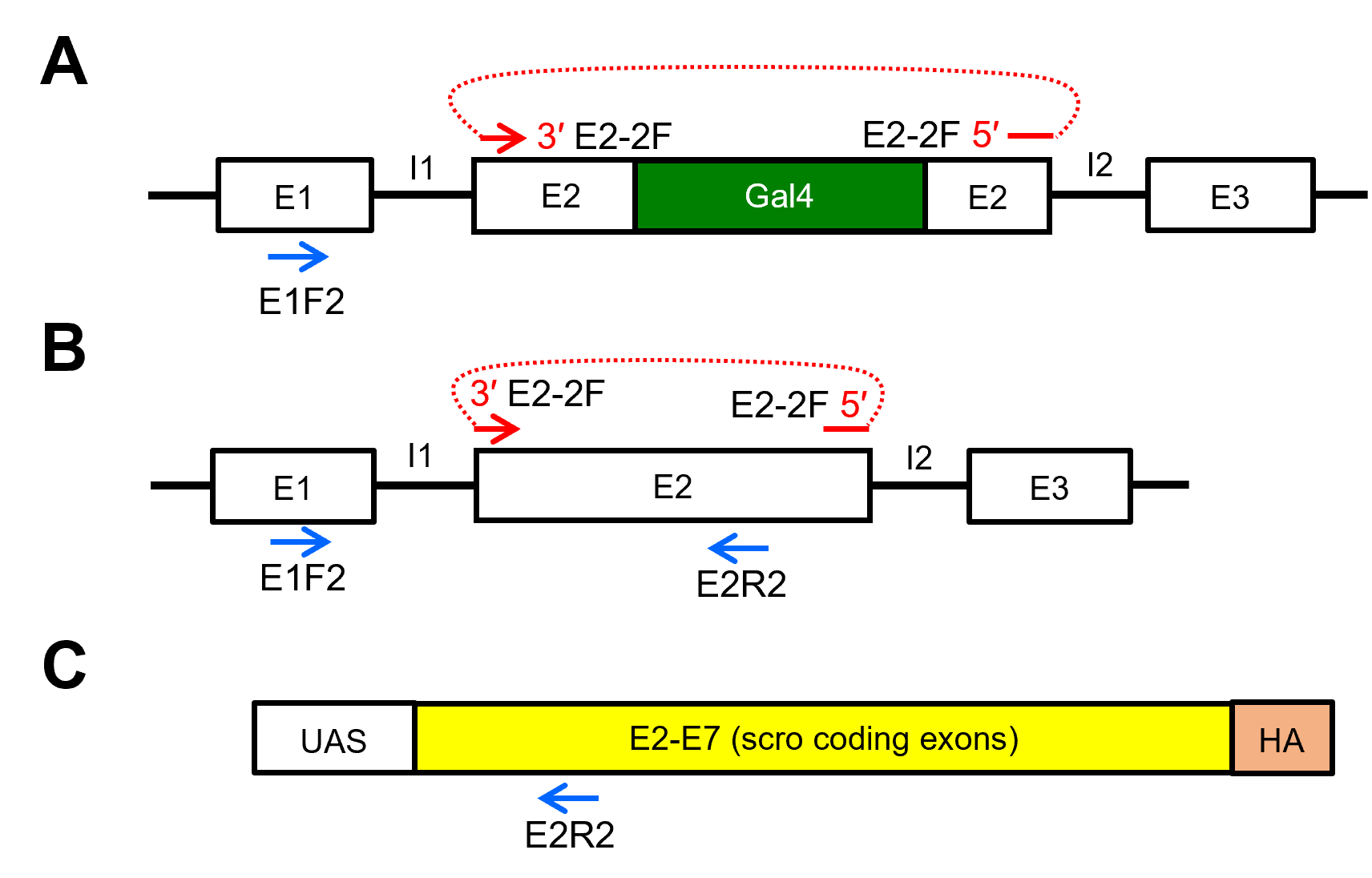

Supplement: jkaf055_Supplementary_Data [file jkaf055_supplementary_data.zip › Supplementary_Figure_6_G3-2025-405711.tif]

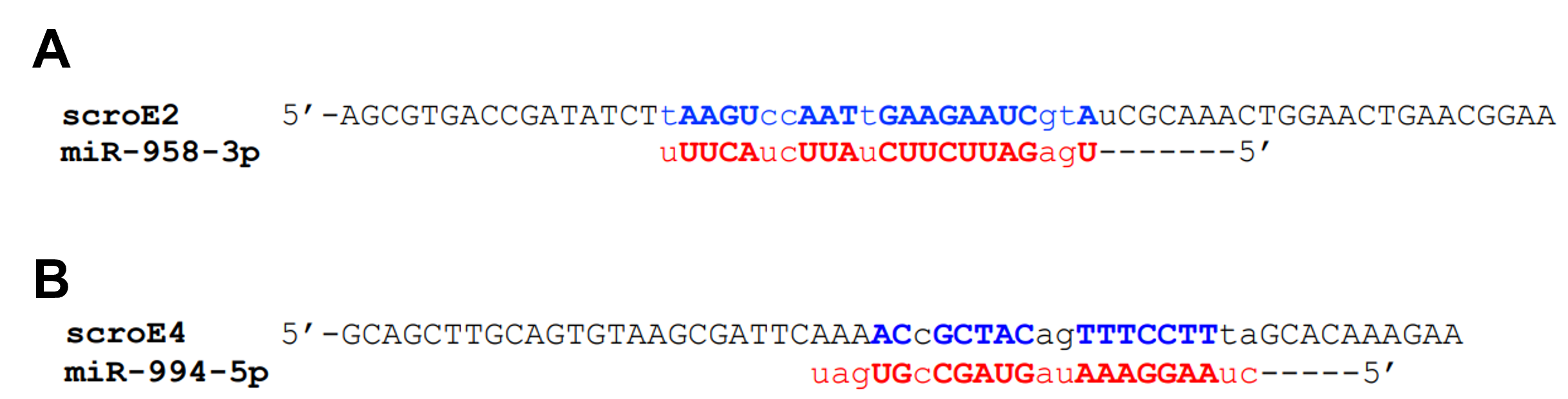

Supplement: jkaf055_Supplementary_Data [file jkaf055_supplementary_data.zip › Supplementary_Figure_7_G3-2025-405711.tif]
